# The Meckel syndrome- associated protein MKS1 functionally interacts with components of the BBSome and IFT complexes to mediate ciliary trafficking and hedgehog signaling

**DOI:** 10.1371/journal.pone.0173399

**Published:** 2017-03-14

**Authors:** Sarah C. Goetz, Fiona Bangs, Chloe L. Barrington, Nicholas Katsanis, Kathryn V. Anderson

**Affiliations:** 1 Program in Developmental Biology, Sloan Kettering Institute, Memorial Sloan Kettering Cancer Center, 1275 York Ave. New York, United States of America; 2 Department of Pharmacology and Cancer Biology, Duke University Medical Center, Durham, NC, United States of America; 3 Department of Cell Biology and Center for Human Disease Modeling, Duke University Medical Center, Durham, NC, United States of America; University of Massachusetts Medical School, UNITED STATES

## Abstract

The importance of primary cilia in human health is underscored by the link between ciliary dysfunction and a group of primarily recessive genetic disorders with overlapping clinical features, now known as ciliopathies. Many of the proteins encoded by ciliopathy-associated genes are components of a handful of multi-protein complexes important for the transport of cargo to the basal body and/or into the cilium. A key question is whether different complexes cooperate in cilia formation, and whether they participate in cilium assembly in conjunction with intraflagellar transport (IFT) proteins. To examine how ciliopathy protein complexes might function together, we have analyzed double mutants of an allele of the Meckel syndrome (MKS) complex protein MKS1 and the BBSome protein BBS4. We find that *Mks1; Bbs4* double mutant mouse embryos exhibit exacerbated defects in Hedgehog (Hh) dependent patterning compared to either single mutant, and die by E14.5. Cells from double mutant embryos exhibit a defect in the trafficking of ARL13B, a ciliary membrane protein, resulting in disrupted ciliary structure and signaling. We also examined the relationship between the MKS complex and IFT proteins by analyzing double mutant between *Mks1* and a hypomorphic allele of the IFTB component *Ift172*. Despite each single mutant surviving until around birth, *Mks1; Ift172*^*avc1*^ double mutants die at mid-gestation, and exhibit a dramatic failure of cilia formation. We also find that *Mks1* interacts genetically with an allele of *Dync2h1*, the IFT retrograde motor. Thus, we have demonstrated that the MKS transition zone complex cooperates with the BBSome to mediate trafficking of specific trans-membrane receptors to the cilium. Moreover, the genetic interaction of *Mks1* with components of IFT machinery suggests that the transition zone complex facilitates IFT to promote cilium assembly and structure.

## Introduction

Its function once mysterious, the primary cilium has become the focus of considerable interest in recent years. A number of human recessive genetic disorders, termed ciliopathies, are caused by mutations in genes encoding proteins that localize to the cilium or to the basal body, the modified centriole that nucleates the cilium [1–3]. At the molecular level, cilia are required for the regulation of the Hh pathway [4–7] as well as in signaling downstream of G-protein coupled receptors (GPCRs) in the nervous system [8–10], implicating cilia in the regulation of vital signal transduction pathways. Ciliopathies are pleiotropic disorders; for many years the mechanisms by which diverse organ systems could be disrupted within individual conditions remained a puzzle. The association of these disorders with the primary cilium has now provided a framework to understand the underlying basis for these diseases. There are currently more than a dozen established ciliopathies, including Bardet Biedl (BBS), nephronophthisis (NPHP), Joubert (JBTS), Meckel-Gruber (MKS), short-rib polydactyly (SRP), and oral-facial digital (OFD) syndromes. These disorders vary in severity and have distinct features, though they often share common elements, most notably renal and hepatic cysts [11]. More recently, ciliary dysfunction has also been postulated to contribute to the etiopathology of more complex disorders, most notably autism and schizophrenia [12–14].

Ciliopathies are also characterized by genetic complexity. While mutations affecting particular genes are most commonly associated with a specific ciliopathy (i.e. *Mks1-3* are associated with Meckel syndrome), different mutations in a single gene have been associated with different ciliopathies [15,16]. In addition, mutations in a subset of *Bbs* genes have been identified in fetuses diagnosed with MKS, and vice versa [16,17]. Individual ciliopathy patients have also been reported with mutations in multiple ciliary and ciliopathy-associated genes, providing evidence for potentially complex additive or multiplicative genetic interactions between ciliopathy loci [18–24]. Understanding the mechanisms by which particular mutations or sets of mutations contribute to specific phenotypes is of central importance for both understanding pathomechanisms and for clinical management.

Cilium assembly is a highly complex process. The assembly of the ciliary axoneme and the transport of proteins into and out of cilia is mediated by the evolutionarily conserved process of IFT. Trafficking of cargo into the cilium is controlled by the kinesin-2 motor, together with two multi-protein complexes, the IFT-A and IFT-B particles. Transport from the tip of the cilium back to the base is mediated by a dedicated cytoplasmic dynein motor [3,25]. In addition to the IFT machinery, four complexes of ciliopathy-associated proteins have been identified that are involved with different aspects of ciliary trafficking: the BBSome, the MKS complex and two NPHP complexes. This has helped to provide clues as to how distinct proteins regulate cilia function [26–28]. Evidence suggests that the BBSome functions as a membrane coat complex that promotes trafficking of specific GPCRs and other proteins to the ciliary membrane [29]. The BBSome or a subset of BBSome components also traffic within cilia and may be associated at sub-stoichiometric levels with IFT complexes [30]. The MKS and NPHP complexes function at the ciliary transition zone between the basal body and axoneme, where they regulate entry and exit of specific membrane proteins to and from the cilium, thus helping to maintain the unique protein composition of the ciliary membrane [31,32].

A key question about the function of the ciliopathy complexes is the nature of their relationships with one another and with IFT in ciliary structure and trafficking. Evidence from *C*. *elegans* and the mouse suggests an evolutionarily conserved cooperation between the MKS and NPHP transition zone modules in mediating ciliary trafficking [33,34]. The MKS complex and BBSome genetically interact in zebrafish [16] and also appear to have overlapping functions in promoting cilia formation and Hh signaling in the mouse (39). In addition to the BBSome and the MKS and NPHP modules at the ciliary transition zone, hypomorphic alleles of components of the IFT complexes are also associated with ciliopathies. Every component of the IFT-A complex has been found to be mutated in human ciliopathies [35], as have a number of IFT-B complex components [36,37].

Given that ciliopathies have both overlapping and unique features, dissecting how the proteins and protein complexes work together can help us to understand ciliogenesis as well as the molecular basis of these genetic disorders. We set out to genetically test whether components of two ciliopathy complexes, the BBSome and MKS complex, function together to mediate cilia formation and whether either complex cooperates with IFT in cilium assembly. We show that MKS1 cooperates with BBS4 to mediate trafficking of subset of membrane proteins to the primary cilium. Moreover, we demonstrate that MKS1 functions together with the IFT machinery to mediate ciliogenesis, while BBS4 apparently does not.

## Methods

### Ethics statement

The use and care of mice as described in this study was approved by the Institutional Animal Care and Use Committees of Memorial Sloan Kettering Cancer Center (approval number 02-06-013) and Duke University (approval number A246-14-10). Euthanasia for the purpose of harvesting embryos was performed by cervical dislocation, and all animal studies were performed in compliance with internationally accepted standards.

### Mouse strains

A previously described *Bbs4* genetrap allele [38], in which a trapping vector was inserted into intron 1, was used to generate *Bbs4*^*-/-*^ embryos. We also made use of the following ENU-induced alleles in our genetic analysis: *Mks1*^*krc*^ [39], *Ift172*^*avc1*^ [40], and *Dync2h1*^*mmi*^ [41]. Genotyping for all alleles was performed as previously reported. Details of the numbers of embryos of each genotype obtained for all crosses is presented in S1 Table.

### Cell culture and immunostaining

MEFs were isolated from Embryonic Day (E)10.5 embryos and prepared and maintained as described [42]. Cells were shifted from 10% to 0.5% FBS the day after plating and maintained in low serum media for 48 hours to induce ciliogenesis. Cells were grown on coverslips and fixed in 4% Paraformaldehyde (PFA) in Phosphate Buffered Saline (PBS) for 5 minutes at room temperature followed by methanol for 5 minutes at -20C and washed extensively in PBS + 0.1% Triton X-100 (PBT). Fixed cells were placed in blocking solution (PBT + 5% FBS + 1% bovine serum albumin) for 30 minutes. Cells were then incubated with primary antibodies diluted in blocking solution overnight at 4°C. Cells were then washed PBT and incubated with Alexa-coupled secondary antibodies and DAPI in blocking solution for 30 minutes at room temperature and affixed to slides for microscopy.

Cross sections of embryos tissues were obtained by fixing embryos in 2% PFA overnight at 4C. Embryos were then cryoprotected in 30% sucrose in PBS followed by embedding in OCT and freezing on dry ice. Transverse sections were cut at a thickness of 12μm onto slides, dried, washed in PBT + 1% serum, and incubated with primary antibodies as described below.

### Antibodies

The SMO antibody was raised in rabbits (Pocono Rabbit Farm and Laboratory Inc.) using antigens and procedures described in Rohatgi *et*. *al*. [43]; and used at a dilution of 1:500. The KIF7 antibody [41] was used at 1:1000. Monoclonal antibodies against NKX2.2 and ISL1 were used at 1:10 and obtained from the Developmental Studies Hybridoma Bank. Commercially available antibodies used were: rabbit α-FOXA2 (Millipore, 1:500) rabbit α-OLIG2 (1:200, Millipore), mouse α-acetylated α-Tubulin (1:5000, Sigma Aldrich), mouse α**-**γ**-**Tubulin (1:5000, Sigma Aldrich), rabbit α-IFT88 (1:500, Proteintech), rabbit α-IFT81 (1:200, Proteintech), INPP5E (1:200, Proteintech), GPR161 (1:100, Proteintech). The GLI2 [44] and ARL13B [45] antibodies were described previously.

### Fluorescence microscopy

Cilia images were obtained using a DeltaVision image restoration microscope (Applied Precision/Olympus) equipped with CoolSnap QE cooled CCD camera (Photometrics). An Olympus 100×/1.40 NA, UPLS Apo oil immersion objective was used. Z-stacks were taken at 0.20-μm intervals. Images were deconvolved using the SoftWoRx software (Applied Precision/DeltaVision), and corrected for chromatic aberrations.

Confocal microscopy was performed using an upright Leica SP8 upright laser scanning microscope. Images were taken with a 63X water objective and 1X zoom. Extended views of the confocal datasets were processed using the Volocity software package (Improvision) or ImageJ.

The percentage of ciliated cells was measured as the proportion of cells with either ARL13b or acetylated α–Tubulin as indicated localized adjacent to a γ-Tubulin positive centrosome in 4 randomly selected fields per slide, averaged across 3 slides per genotype. Fields were imaged using a Zeiss Axioplan2 epifluorescence microscope. Cells were counted using ImageJ and data was analyzed with GraphPad Prism 6 software, using a one-way ANOVA with a Tukey-Kramer post-hoc test for significance.

### Scanning electron microscopy

E10.5 embryos were dissected in PBS at RT and immediately fixed in 2.5% glutaraldehyde and 2% PFA in 0.1M sodium cacodylate buffer, pH 7.4 (Electron Microscopy Sciences) overnight at 4°C. The neural tube of the embryo at the level of the forelimbs was then separated from surrounding tissue and divided into two lateral halves. This tissue was then stored in 0.1M sodium cacodylate buffer overnight, followed by dehydration, drying and coating as previously described [4]. Scanning electron micrograph images were taken on a Zeiss SUPRA 25 FESEM.

### qPCR

The MEFs derived from embryos of each genotype indicated were allowed to reach confluence and serum-starved from for 48 hours. The cells were treated with SAG (100nM) or DMSO for the last 24 hours of serum starvation. Each genotype and condition was plated in triplicate. The RNA was extracted using RNeasy Mini Kit (Qiagen) following manufacturer’s instructions, with DNase treatment. Following RNA extraction, cDNA was produced with the iScript cDNA Synthesis Kit (BioRad). RT-qPCR was prepared with Taqman probes for Gli (Thermo, Mm00494654_ml) and housekeeping gene GAPDH (Thermo, Mm99999915_g1). Three technical replicates were performed for each biological replicate, using 9.8 ng of cDNA each, and an accompanying water control. The RT-qPCR was performed as a 96-well standard, using Applied Biosystems 7900HT Real-Time PCR System. Thermal cycling conditions are specified in Taqman Gene Expression Assays Protocol.

## Results

### BBSome and MKS complex components genetically interact

**Fig 1:**
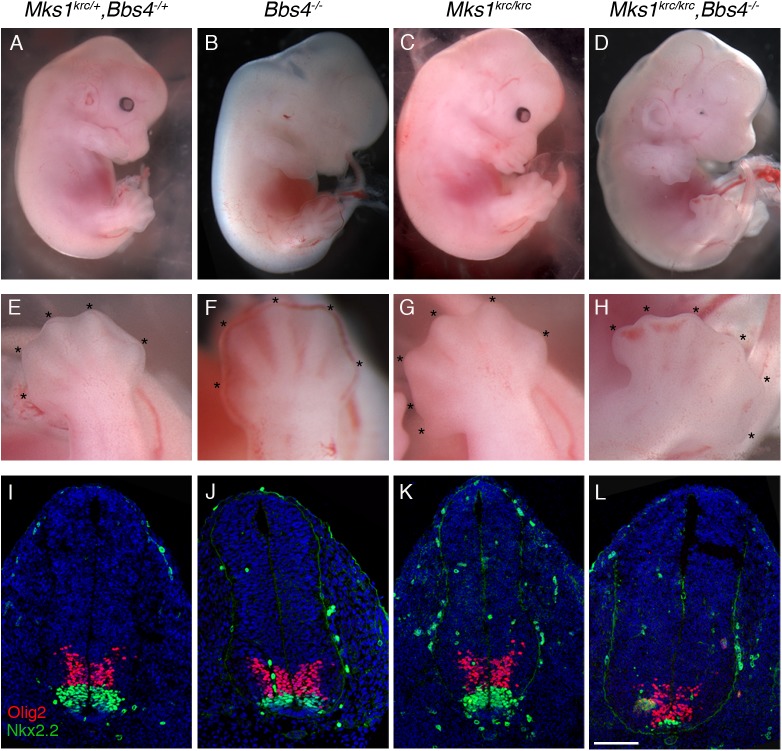
Loss of *Bbs4* enhances Hh signaling phenotypes in *Mks1*^*krc*^ mutant embryos. **(A-D)** Double heterozygous (A), *Bbs4*^*-/-*^ (B), *Mks1*^*krc/krc*^ (C), or *Mks1*^*krc/krc*^*;Bbs4*^*-/-*^ double mutant (D) embryos at E13.5. Note that double mutant embryos exhibit microopthalmia, polydactyly, and general edema. **(E-H)** Higher magnification images of the hindlimbs of the embryos indicated above. Each digit is denoted by *. Approximately 25% of *Mks1*^*krc/krc*^ exhibit polydactyly on at least one limb as in (G). Double mutant embryos exhibit fully penetrant polydactyly on all four limbs (H). **(I-L)** Transverse sections through the neural tube of embryos of the indicated genotype at E10.5. Sections were taken at the level of the forelimbs and immunostained with antibodies against OLIG2 (red) and NKX2.2 (green). Scale bar, 100μm.

BBS and MKS syndromes display phenotypic overlap in human patients [17]. To examine whether MKS1 and the MKS complex have roles in ciliary trafficking that may parallel those of other ciliopathy complexes, we tested whether *Mks1* interacts genetically with the BBSome component *Bbs4*. We generated *Mks1; Bbs4* double mutant embryos, using the previously described ENU-induced *Mks1*^*krc*^ allele [39] and a *Bbs4* genetrap allele (here referred to as *Bbs4*^*-/-*^) [38]. The *Mks1*^*krc*^ allele harbors a point mutation within a splice donor site that causes an insertion and an in-frame stop codon. Due to the lack of any WT splicing and early truncation of the protein caused by this mutation, *Mks*^*krc*^ is thought to be a null allele of *Mks1* [39]. We confirmed that another component of the MKS complex, TMEM67, fails to localize to the transition zone of *Mks1*^*krc/krc*^ cells, consistent with reports indicating that the individual components of the MKS complex are required for its stability [31] (S1 Fig). At E13.5, *Bbs4*^*-/-*^ embryos were indistinguishable from wild-type siblings, as were *Mks1*^*krc*^ mutant embryos, with the exception of pre-axial polydactyly occurring in about 25% of these embryos (n = 33, 8 exhibited polydactyly) due to disrupted Hh signaling in the limb [39] (Fig 1A–1C). By contrast, *Bbs4*^*-/-*^*; Mks1*^*krc/krc*^ double mutant embryos were never recovered after E14.5 and displayed microopthalmia and pronounced edema (Fig 1D). Moreover, while pre-axial polydactyly is seen in a fraction of *Mks1*^*krc/krc*^ mutants as a duplication of digit 1 on at least one limb [39] (Fig 1G), 100% of double mutant embryos examined at E13.5 or E14.5 exhibited a duplication of digit 1 on all four limbs (n = 18; Fig 1D and 1H; summarized in Table 1).

**Table 1:** Summary of embryonic phenotypes in double mutant crosses.

Mks1^krc^ x Bbs4^-/-^				
Genotype	Mks1^krc/+^; Bbs4^+/-^	Mks1^krc/krc^	Bbs4^-/-^	Mks1^krc/krc^; Bbs4^-/-^
Embryonic Lethal?	No	Yes. Variable: E13.5-E17.5[39]	No	Yes. E13.5
Neural Patterning	Normal	Slight alterations: dorsal expansion of motor neurons, reduction of Nkx2.2. Varies along A-P axis[39]	Normal	Reduced Nkx2.2, ventral shift of motor neurons.
Limbs	Normal	Pre-axial polydactyly in ~50% on at least 1 limb	Normal	Polydactyly in all embryos, all 4 limbs
Cilia Number	Normal	Reduced by ~60% compared to WT	Normal	Similar to Mks1^krc/krc^. Lack of ARL13b and other cilia membrane proteins
Mks^krc^ x Ift172^avc1^				
Genotype	Mks1^krc/+^; Ift172^avc1/+^	Mks1^krc/krc^	Ift172^avc1/avc1^	Mks1^krc/krc^; Ift172^avc1/avc1^
Embryonic Lethal?	No	Yes. Variable: E13.5-E17.5[39]	Perinatal lethal	Yes. E10.5
Neural Patterning	Normal	Slight alterations: dorsal expansion of motor neurons, reduction of Nkx2.2. Varies along A-P axis[39]	Normal in brachial spinal cord, in lumbar region, motor neurons expanded dorsally [46]	Loss of ventral cell types, motor neurons strongly reduced
Limbs	Normal	Pre-axial polydactyly in ~50% on at least 1 limb	Fully penetrant forelimb polydactyly[40]	N/A (Embryos die prior to digit formation.)
Cilia Number	Normal	Reduced by ~50–60% compared to WT	Similar to WT	Strongly reduced.
Mks1^krc^ x Dync2h1^mmi^				
Genotype	Mks1^krc/+^; Dync2h1^mmi/+^	Mks1^krc/krc^	Dync2h1^mmi/mmi^	Mks1^krc/krc^; Dync2h1^mmi/mmi^
Embryonic Lethal?	No	Yes. Variable: E13.5-E17.5[39]	Yes, E10.5	Yes, E10.5
Neural Patterning	Normal	Slight alterations: dorsal expansion of motor neurons, reduction of Nkx2.2. Varies along A-P axis [39].	Loss of ventral cell types, motor neurons present but reduced.	Loss of ventral cell types, motor neurons strongly reduced.
Cilia Number	Normal	Reduced by ~60% compared to WT	Similar to WT, with structural abnormalities.	Strongly reduced.
Bbs4^-/-^ x Ift172^avc1^				
Genotype	Bbs4^+/-^; Ift172^avc1/+^	Bbs4^-/-^	Ift172^avc1/avc1^	Bbs4^-/-^; Ift172^avc1/avc1^
Embryonic Lethal?	No	No	Perinatal lethal	Perinatal lethal
Neural Patterning	Normal	Normal	Normal in brachial spinal cord, in lumbar region, motor neurons expanded dorsally [46]	Similar to Ift172^avc1/avc1^ single mutants
Cilia Number	Normal	Normal	Similar to WT	Similar to WT

A well-characterized role for non-motile primary cilia in the developing embryo is in mediating Hh signaling; perturbations to primary cilia structure or function lead to disruptions of Hh-dependent patterning in a variety of tissues, including the developing limbs and spinal cord [4–6,45]. To determine whether the enhanced severity of the double mutant embryos compared with *Mks1*^*krc/krc*^ or *Bbs4*^*-/-*^ single mutants were due to exacerbated defects in Hh signaling, we examined ventral neural patterning in *Bbs4*^*-/-*^*; Mks1*^*krc*^, and double mutant embryos. V3 interneuron progenitors, labeled by NKX2.2, depend on high levels of Sonic Hedgehog (SHH) from the notochord and floorplate for their specification. Motor neuron progenitors (labeled by OLIG2) depend on intermediate levels of SHH signal [47]. We examined both cell types at the forelimb level within the neural tube as a read-out of Hh pathway activity in double mutants and *Bbs4* and *Mks1* single mutants at E10.5. *Bbs4*^*-/-*^ mutant embryos exhibit normal neural patterning (Fig 1J). Previous studies examining the phenotypes of *Mks1* mutant embryos report complex neural patterning phenotypes wherein cell types that require intermediate levels of SHH for their patterning are slightly expanded, with the ventral most cells including V3 interneurons slightly reduced in number in more caudal regions of the neural tube [39,48]. We similarly observed only a slight expansion of OLIG2+ motor neuron progenitors at the forelimb level in *Mks1* single mutants (Fig 1K). By contrast, in double mutants NKX2.2+ cells are dramatically reduced and motor neurons have shifted ventrally to span the ventral midline (Fig 1L). Taken together, these phenotypes suggest that removal of *Bbs4* in a *Mks1* mutant background exacerbates the Hh- dependent patterning defects observed in *Mks1*^*krc/krc*^ embryos. We also tested the responsiveness of cells derived from *Bbs4*^*-/-*^*; Mks1*^*krc/krc*^ double mutant embryos compared with WT and single mutant cells by performing qPCR for *Gli1* levels in response to addition of SMO agonist (SAG), a potent activator of Hh signaling (S2 Fig). We find that both *Bbs4*^*-/-*^ and *Mks1*^*krc/krc*^ single mutant fibroblasts exhibited an increase in *Gli1* relative to WT cells. Consistent with the enhanced phenotypes seen in double mutant embryos, *Bbs4*^*-/-*^*; Mks1*^*krc/krc*^ cells exhibit a dampened response to SAG as measured by *Gli1* transcript levels, despite the increase in *Gli1* in each single mutant.

While *Mks1*^*krc/krc*^ embryos do form cilia in most cell types, these cilia are sparse and shorter than wild-type [39]. To determine whether the enhanced severity of the Hh-related phenotypes in double mutant embryos is due to a greater disruption in ciliogenesis we examined cilia formation in double mutant embryos compared with single *Bbs4*^*-/-*^ or *Mks1*^*krc/krc*^ mutants and wild-type sibling embryos.

**Fig 2:**
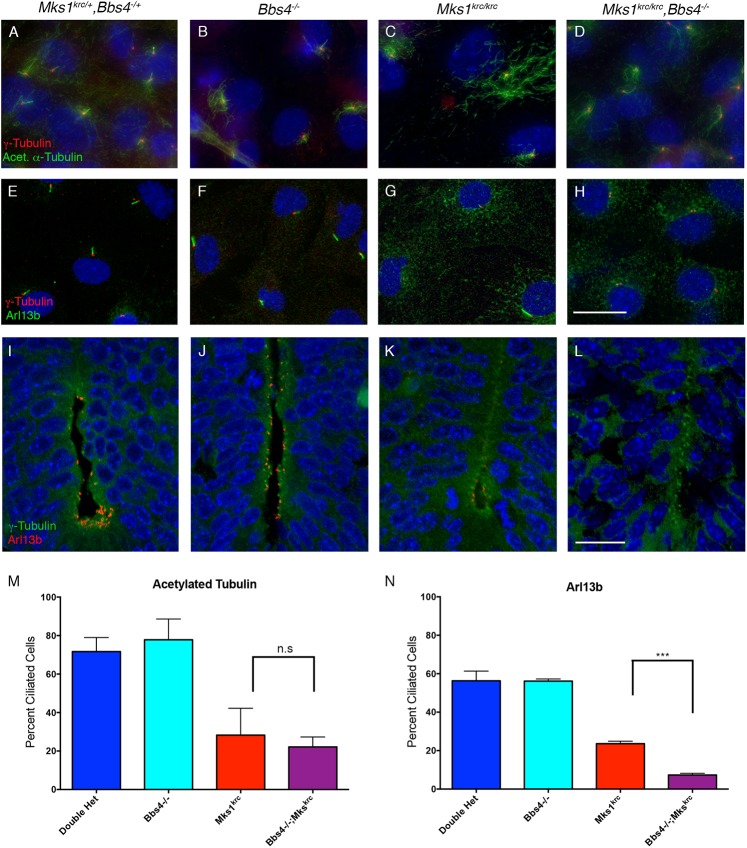
*Mks1*^*krc/krc*^*;Bbs4*^*-/-*^ double mutants exhibit enhanced defects in ciliary localization of Arl13b, but not acetylated Tubulin. **(A-H)** Mouse embryonic fibroblasts (MEFs) were derived from embryos of the indicated genotype, and immunostained for γ-Tubulin (red) and either acetylated α–Tubulin (A-D, green) or ARL13B (E-H, green) to label cilia. Scale bar, 20μm. **(I-L)** Tissue sections from the embryonic neural tube of double heterozygous (A) *Bbs4*^*-/-*^ (B), *Mks1*^*krc/krc*^ (C), or *Mks1*^*krc/krc*^*;Bbs4*^*-/-*^ double mutant (D) at E10.5. Sections were immunostained for ARL13B to label ciliary membranes (red) and for γ-Tubulin to label centrioles (green). Scale bar, 50μm. **(M-N)** Quantitation of the percentage of ciliated cells in each genotype positive for either acetylated α-Tubulin (M) or ARL13B (N) following 48 hours of serum starvation. The percentage of cells with cilia stained for acetylated α–Tubulin was not different between double mutants and *Mks1*^*krc/krc*^ single mutant cells (M, p = 0.3493). By contrast, significantly fewer double mutant cells had cilia stained with ARL13B compared with *Mks1*^*krc/krc*^ single mutant cells (N, *** p = 0.0001).

Mouse embryonic fibroblasts (MEFs) were derived from embryos of each genotype and immunostained for acetylated α-Tubulin, which labels the stable microtubules of the ciliary axoneme. We found that primary ciliogenesis is unaffected in *Bbs4*^*-/-*^ mutant cells compared with heterozygous controls (Fig 2A, 2B and 2M), while cilia are noticeably sparse in *Mks1*^*krc/krc*^ mutant cells (Fig 2C and 2M), consistent with previous studies [38,49]. *Bbs4*^*-/-*^*; Mks1*^*krc/krc*^ double mutant cells had comparable numbers of cilia to those observed in *Mks1*^*krc/krc*^ single mutants (Fig 2C, 2D and 2M). SEM performed on embryonic neural tubes at E10.5 also revealed similar numbers and appearance of cilia between *Bbs4*^*-/-*^*; Mks1*^*krc/krc*^ double mutant and in *Mks1*^*krc/krc*^ single mutant embryos (S3 Fig).

In contrast, immunostaining with an antibody against the ciliary membrane protein ARL13B in both embryonic tissues and MEFs revealed a dramatic defect in *Bbs4*^*-/-*^*; Mks1*^*krc/krc*^ double mutants. In both double heterozygous and *Bbs4*^*-/-*^ MEFs, over half of the cells formed an ARL13B+ cilium (56.3% and 56.1%; n>200 for each condition; Fig 2E, 2F and 2N), this number was reduced to a mean of 23.6% in *Mks1*^*krc/krc*^ cells (n = 420 cells, Fig 2G and 2N). Strikingly, even though *Bbs4*^*-/-*^ cells form cilia at a rate nearly identical to that of heterozygotes, *Bbs4*^*-/-*^*; Mks1*^*krc/krc*^ double mutant cells had a more severe defect in ARL13B trafficking than *Mks1*^*krc/krc*^ mutant cells, with only 7.3% of double mutant cells forming an identifiable ARL13B+ cilium (n = 375 cells, Fig 2H and 2N). In support of our findings from MEFs, similar defects in ARL13B are observed upon immunostaining of the embryonic neural tube at E10.5 (Fig 2I–2L). Therefore, while cells of *Bbs4*^*-/-*^*; Mks1*^*krc/krc*^ double mutants have ciliated cells at similar frequencies to those of *Mks1*^*krc/krc*^ mutants, the loss of *Bbs4* in *Mks1*^*krc/krc*^ mutants results in additional defect in trafficking of the ciliary membrane protein, ARL13B. Based on this finding, we hypothesized that the BBSome and MKS complexes cooperate in the trafficking of specific ciliary membrane proteins.

### BBS4 and MKS1 regulate transport of a subset of ciliary membrane proteins

**Fig 3:**
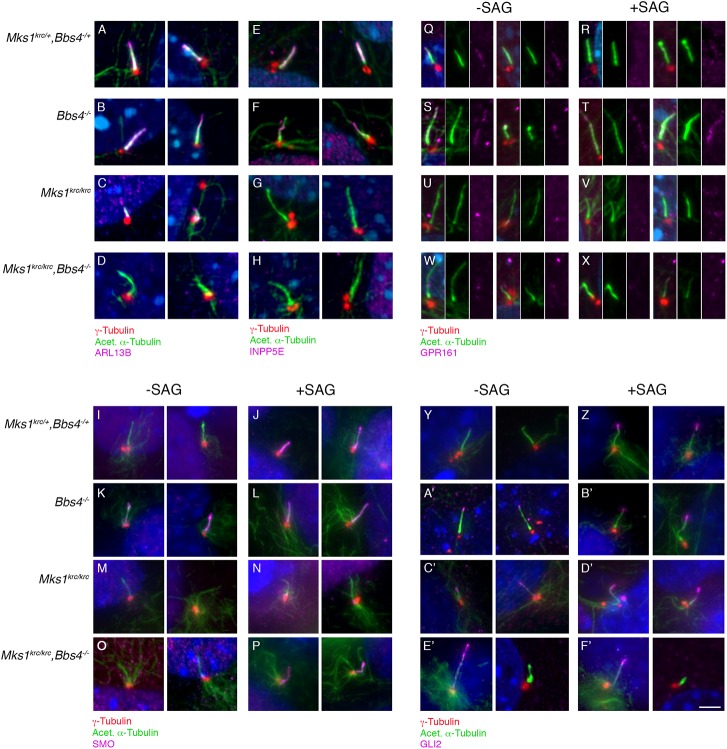
Ciliary trafficking defects in *Mks1*^*krc/krc*^*;Bbs4*^*-/-*^ double mutant cells. **(A-H)** MEFs derived from embryos of the indicated genotypes were cultured under serum-free conditions for 48 hours to induce cilia formation and immunostained for γ-Tubulin (red), acetylated α–Tubulin (green) and the ciliary membrane proteins ARL13B (A-D, magenta) or INPP5E (E-H, magenta). Two representative images for each condition are included. **(I-P)** Serum starved MEFs derived from embryos of each genotype were treated for the final 24 hours with either DMSO vehicle (I,K,M,O) or SMO agonist (SAG) to activate the Hh pathway (J,L,N,P). Cells were stained for γ-Tubulin (red), acetylated α–Tubulin (green), or SMO (magenta). Two representative images for each condition are included. **(Q-X)** MEFs of each genotype were treated as described for (I-P) and immunostained for γ-Tubulin (red), acetylated α–Tubulin (green), or GPR161. Due to the low intensity of GPR161 relative to acetylated α–Tubulin, each of these channels is shown separately next to the corresponding merged channel image. Two representative images for each condition are included. **(Y-F’)** MEFs of each genotype were treated as described in I-X and immunostained for α–Tubulin (red), acetylated α-Tubulin (green), or GLI2 (magenta). Two representative images for each condition are included. Scale bar, 5μm.

To confirm that the reduced proportion of ARL13B+ cells in *Bbs4*^*-/-*^*; Mks1*^*krc/krc*^ double mutants relative to the overall numbers of ciliated cells counted is due specifically to a failure of ARL13B to localize to cilia that are present, we performed double labeling for ARL13B and acetylated α-Tubulin. While in cells from double heterozygous, *Bbs4*^*-/-*^, and *Mks1*^*krc/krc*^ embryos, each axoneme recognized by acetylated α-Tubulin was also positive for ARL13B (Fig 3A–3C), in *Bbs4*^*-/-*^*; Mks1*^*krc/krc*^ double mutants, nearly all cilia labeled by acetylated α-Tubulin lacked ARL13B (Fig 3D). This is consistent with our data quantifying the percentage of cells with each ciliary marker and shows that *Bbs4*^*-/-*^*; Mks1*^*krc/krc*^ have exacerbated defects in the trafficking of this ciliary membrane protein.

To build upon this finding, we also examined a variety of different proteins that localize to the ciliary membrane to mediate cell signaling. These include INPP5E, a lipid phosphatase that helps to regulate the trafficking of other membrane proteins into and out of the cilium [50,51] as well as SMO and GPR161, G-protein coupled receptors (GPCRs) integral to the Hh pathway [50–52].

INPP5E binds to ARL13B, and this interaction has been shown to facilitate the trafficking of INPP5E into cilia [53]. Given this association, and our finding that ARL13B trafficking defects were further enhanced in *Bbs4*^*-/-*^*; Mks1*^*krc/krc*^ double mutants compared with *Bbs4*^*-/-*^ or *Mks1*^*krc/krc*^, we examined INPP5E localization to cilia in these mutants (Fig 3E–3H, S4 Fig). In double heterozygous cells, we found that a mean of 64.5% of cilia were positive for INPP5E (n = 219). In *Bbs4*^*-/-*^ cells, this proportion was significantly reduced (p<0.0001) 37.4% of cilia were INPP5E positive (n = 88). However, we found that there was no detectable INPP5E in either *Mks1*^*krc/krc*^ mutant cilia (n = 33 cells, Fig 3G), or in the cilia of in *Bbs4*^*-/-*^*; Mks1*^*krc/krc*^ double mutant cells (n = 25 cells, Fig 3H), consistent with a requirement for MKS1 in the trafficking of INPP5E to cilia.

SMO is a GPCR-related protein that functions as an upstream activator of Hh signaling and localizes to cilia. SMO becomes enriched in cilia upon stimulation of the pathway with either SHH or the SMO agonist, SAG [54]. Consistent with previous reports [43,54], we find that SMO becomes more highly enriched in the ciliary membrane in double heterozygous cells upon treatment with SAG (-SAG: 38.1% cilia SMO+, n = 49; +SAG: 84.5% cilia SMO+, n = 40; Fig 3I and 3J). In contrast, in *Bbs4*^*-/-*^ cells SMO is enriched in the ciliary membrane in the absence of SAG stimulation (-SAG: 64.1% cilia SMO+, n = 38; +SAG: 83.8% cilia SMO+, n = 65; Fig 3K and 3L), similar to reports for other BBSome mutants [55], while in *Mks1*^*krc/krc*^ cells, SMO accumulation in the cilium is dampened (-SAG: 12.7% cilia SMO+, n = 44; +SAG: 32.2% cilia SMO+, n = 38; Fig 3M and 3N). Accumulation of SMO in cilia upon stimulation with SAG is restored in *Bbs4*^*-/-*^*; Mks1*^*krc/krc*^ double mutant MEFs, (-SAG: 19.4% cilia SMO+, n = 26; +SAG: 82.4% cilia SMO+, n = 38; Fig 3O and 3P), indicating that loss of *Bbs4* is sufficient to overcome the dampening of SMO accumulation in response to SAG that we observe in the *Mks1*^*krc/*krc^ mutant cells. The graph quantifying this data can be seen in S4 Fig.

We also examined trafficking of GPR161 in response to Hh pathway activation in each of the mutants. GPR161 is a GPCR that acts as a negative regulator of the Hh pathway. Consistent with its role in suppressing Hh signaling, GPR161 is present in cilia in the absence of pathway stimulation, and is lost from cilia when cells are treated with SHH or SAG to activate the pathway [52]. We observed this pattern of localization in double heterozygous cells (-SAG: 70.8% cilia GPR161+, n = 68; +SAG: 35.8% cilia GPR161+, n = 52; Fig 3Q and 3R, graph of quantification S4 Fig), while in *Bbs4*^*-/-*^ mutant cells, GPR161 is detected in cilia even when the cells are exposed to SAG (-SAG: 78.9% cilia GPR161+, n = 72; +SAG: 75.9% cilia GPR161+, n = 54; Fig 3S and 3T). In *Mks1*^*krc/krc*^ single mutant cells, GPR161 was detected in cilia only at low frequency in the absence of pathway activation, and was entirely lost from cilia in cells treated with SAG (-SAG: 8.0% cilia GPR161+, n = 30; +SAG: 0% cilia GPR161+, n = 48; Fig 3U and 3V), suggesting that MKS1 is essential for trafficking of GPR161 into cilia. Surprisingly, in *Bbs4*^*-/-*^*; Mks1*^*krc/krc*^ double mutant cells, GPR161 was seen at higher frequencies in cilia compared to *Mks1*^*krc/krc*^ single mutant cells, and the frequency of GPR161 localization to cilia did not change in the double mutant cells upon addition of SAG (-SAG: 21.4% cilia GPR161+, n = 34; +SAG: 29.0% cilia GPR161+, n = 55; Fig 3W and 3X). Together, our SMO and GPR161 data indicate that the enhanced phenotype of *Bbs4*^*-/-*^*; Mks1*^*krc/krc*^ double mutants is the consequence of combined trafficking defects arising from the dual loss of MKS and BBsome function. This indicates that these complexes act in parallel to regulate ciliary membrane composition.

### Bbs4; Mks1 double mutants exhibit disrupted trafficking of the Hh pathway effector GLI2

To test more directly how the disrupted ciliary trafficking observed in the double mutant embryos causes the exacerbated Hh-related phenotypes observed in these embryos, we examined the trafficking of GLI2, the major positive effector of the Hh pathway [54]. Consistent with previous reports, we find that GLI2 becomes enriched at the ciliary tip in response to treatment with SAG in double heterozygous MEFs (-SAG 34.5% cilia GLI2+, n = 79; +SAG 74.9% cilia GLI2+, n = 119, Fig 3Y and 3Z; quantification of GLI2 data in S4 Fig). In *Bbs4*^*-/-*^ cells, a high proportion of both unstimulated as well as stimulated cells have GLI2 at the ciliary tip (-SAG 80.6% cilia GLI2+, n = 78; +SAG 90.5% cilia GLI2+, n = 52; Fig 3A’ and 3B’). In contrast, we found that in *Mks1*^*krc/krc*^ mutant cells, GLI2 was no longer confined to the ciliary tip but was present throughout the distal portion of the axoneme in nearly half of cilia examined (48.9%, S4 Fig), and like *Bbs4*^*-/-*^ cells, GLI2 was seen at fairly high levels in cilia in the absence of SAG (Fig 3C’), and ciliary GLI2 did not increase upon addition of SAG (Fig 3D’; -SAG 88.2% cilia GLI2+, n = 34; +SAG 79.0% cilia GLI2+, n = 36). In *Bbs4*^*-/-*^*;Mks1*^*krc/krc*^ double mutant MEFs, mis-localization of GLI2 within the axoneme is evident (Fig 3E’ and 3F’, S4 Fig), however the response of the double mutant cells to stimulation with SAG was dampened in these cells, and proportion of GLI2+ cilia was comparable to that double heterozygous unstimulated cells, in either the presence or absence of SAG (-SAG 38.1% cilia GLI2+, n = 35; +SAG 45.5% cilia GLI2+, n = 44; S4 Fig). We propose that the combination of perturbed ciliary membrane composition and disrupted trafficking of GLI2 along the axoneme causes the exacerbated Hh-related phenotypes observed in double mutant embryos, and the loss of Hh responsiveness seen in double mutant fibroblasts.

### MKS1 cooperates with IFT to mediate cilium assembly

Through our examination of the trafficking of Hh components in response to pathway activation in *Mks1*^*krc/krc*^, *Bbs4*^*-/-*^, and double mutants, we identified a previously unreported requirement for MKS1 in localizing GLI2 to the ciliary tip. We found that GLI2 is present throughout the distal portion of the axoneme in these mutants (Fig 3C’ and 3D’), reminiscent of other mutants in which retrograde IFT trafficking or ciliary tip structure is disrupted [46,56]. We therefore tested whether the MKS transition zone complex cooperates with IFT by assessing whether MKS1 genetically interacts with components of the trafficking machinery.

**Fig 4:**
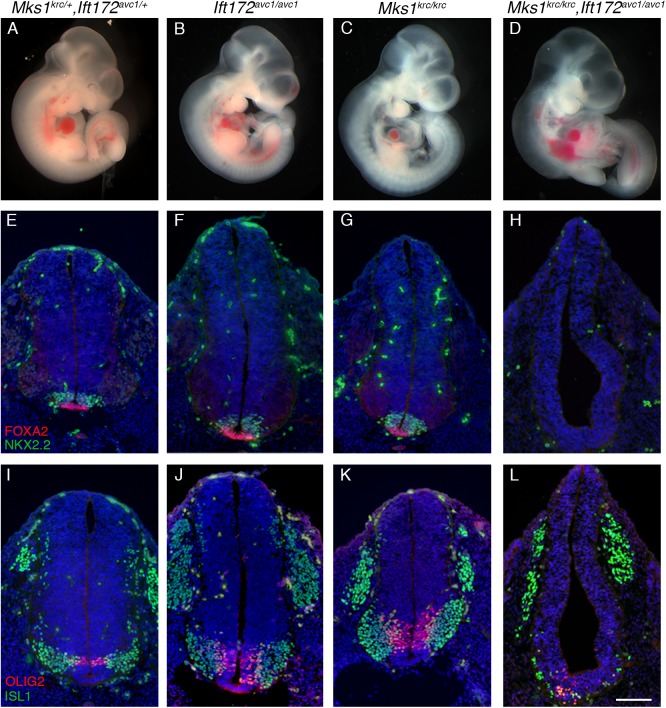
*Mks1*^*krc/krc*^*;Ift172*^*avc1/avc1*^ double mutants exhibit phenotypes consistent with severely reduced Hh signaling. **(A-D)** Double heterozygous (A), *Ift172*^*avc1/avc1*^ (B), *Mks1*^*krc/krc*^ (C), or *Mks1*^*krc/krc*^*;Ift172*^*avc1/avc1*^ double mutant (D) embryos at E10.5. In contrast to *Ift172*^*avc1/avc1*^ or *Mks1*^*krc/krc*^ single mutant embryos, double mutants exhibit a pointed midbrain flexure, enlarged branchial arches, and a twisted body axis reminiscent of other mutants in which cilia are absent or ciliogenesis is severely perturbed. **(E-L)** Transverse sections through the neural tube of embryos of the indicated genotype at E10.5. Sections were taken at the level of the forelimbs and immunostained with antibodies against FOXA2 (red) and NKX2.2 (green) in E-H, or OLIG2 (red) and ISL1 (green) in I-L. Scale bar, 100μm.

We first generated double mutants between *Mks1*^*krc*^ and a previously described hypomorphic allele of the IFT-B gene *Ift172*: *Ift172*^*avc1*^ (phenotypes summarized in Table 1) [40]. *Ift172*^*avc1/avc1*^ homozygotes die perinatally, similar to *Mks1*^*krc/krc*^ embryos, however we found that *Mks1*^*krc/krc*^*; Ift172*^*avc1/avc1*^ double mutant embryos die by E10.5 with severe Hh-related patterning defects (Fig 4). At E10.5, *Mks1*^*krc/krc*^ and *Ift172*^*avc1/avc1*^ single mutants have an overall appearance very similar to WT or double heterozygous embryos (Fig 4A–4C), however double mutant embryos show a number of defects, including enlarged branchial arches, a narrowed midbrain flexure, and twisted posterior axis, that are similar to phenotypes seen in other embryos that lack cilia or have severely perturbed ciliary architecture [4–6,57,58] (Fig 4D). Similarly, the patterning of ventral neural progenitors is severely perturbed in *Mks1*^*krc/krc*^*; Ift172*^*avc1/avc1*^ double mutants. Cells of the floorplate, labeled by FOXA2, as well as V3 interneuron progenitors, labeled by NKX2.2 depend on high levels of SHH for their specification and patterning. These cell types are patterned normally in both *Mks1*^*krc/krc*^ and *Ift172*^*avc1/avc1*^ single mutants at this stage (Fig 4E–4G). In contrast, both of these cell types are entirely absent in double mutant embryos (Fig 4H). Motor neuron progenitors, labeled by OLIG2, and differentiated motor neurons, labeled by ISL1, are specified by intermediate levels of SHH. These are dramatically reduced in number in the double mutant embryos, and are shifted ventrally, spanning the midline of the neural tube (Fig 4I–4L). This dramatic enhancement of the severity of the Hh-dependent patterning phenotypes in the double mutant embryos suggests that MKS1 cooperates with the IFT-B complex to facilitate cilia formation. We also examined Hh-responsiveness in MEFs derived from embryos of each genotype by performing qPCR to assess *Gli1* expression in cells in the presence or absence of SAG, as described above for our analysis of *Mks1*^*krc/krc*^*;Bbs4*^*-/-*^ cells. We observed that *Ift172*^*avc1/avc1*^ single mutants activate some expression of *Gli1* in response to SAG, but double mutant cells fail to respond to SAG (S2 Fig).

**Fig 5:**
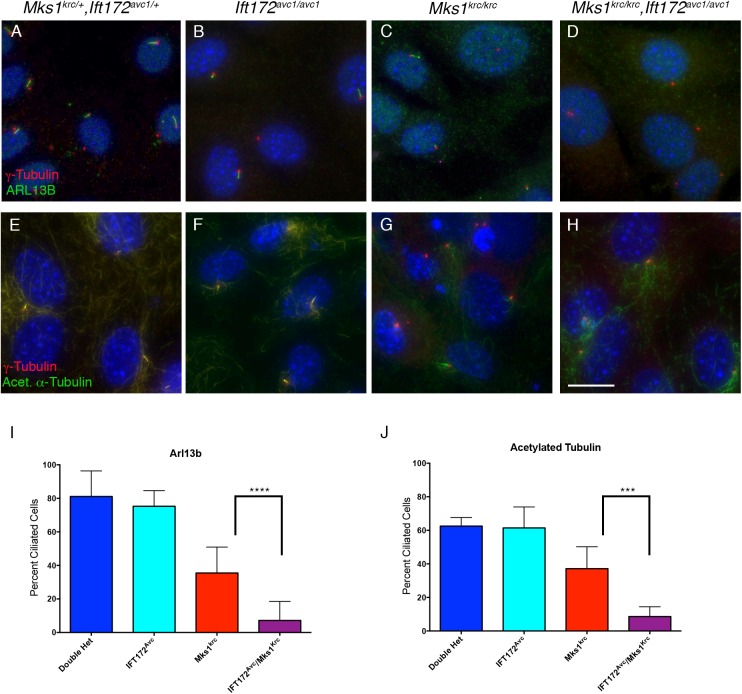
*Mks1*^*krc/krc*^*;Ift172*^*avc1/avc1*^ double mutants exhibit a severe general reduction in ciliogenesis. **(A-H)** MEFs were derived from embryos of the indicated genotype, and immunostained for γ-Tubulin (red) and either ARL13B (A-D, green), or acetylated α-Tubulin (E-H, green). Scale bar, 20μm. **(I-J)** Quantitation of the percentage of ciliated cells in each genotype positive for either Arl13b (I) or acetylated α-Tubulin (J) following 48 hours of serum starvation. Compared with *Mks1*^*krc/krc*^ single mutant cells, significantly fewer double mutant cilia were stained with either ARL13B (I, **** p< 0.0001) or acetylated α-Tubulin (J, *** p = 0.0002). Quantification for each genotype was based on imaging of 4 separate fields on 3 coverslips per condition.

To directly examine whether MKS1 and IFT172 function together to mediate cilium assembly, we derived MEFs from embryos of each genotype and examined the percentage of ciliated cells upon serum withdrawal, using antibodies against ARL13B as well as acetylated α-Tubulin (Fig 5). Cells derived from *Ift172*^*avc1/avc1*^ single mutants had comparable numbers of ciliated cells to double heterozygous MEFs (Double heterozygous: 81.1% ciliated with ARL13b, n = 116, 62.5% ciliated with acetylated α-Tubulin, n = 76; *Ift172*^*avc1/avc1*^: 75.3% ciliated with ARL13b, n = 100, 61.4% ciliated with acetylated α-Tubulin, n-67; Fig 5A, 5B, 5E, 5F, 5I and 5J), while *Mks1*^*krc/krc*^ MEFs exhibited reduced cilia frequencies (35.5% ciliated with ARL13b, n = 230, 37.1% ciliated with acetylated α-Tubulin, n = 85; Fig 5C, 5G, 5I and 5J). Consistent with the enhanced phenotypes related to reduced Hh signaling that we observed in double mutant embryos, we find that cells derived from *Mks1*^*krc/krc*^*; Ift172*^*avc1/avc1*^ mutants have very few cilia labeled with either ARL13B (7.2%, n = 114; Fig 5D and 5I) or acetylated α-Tubulin (8.6%, n = 69; Fig 5H and 5J). We confirmed that the double mutants almost entirely lack cilia by SEM on the embryonic neural tube (S3 Fig). Notably, this failure of ciliogenesis contrasts with the defect we observed in the *Bbs4*^*-/-*^*;Mks1*^*krc/krc*^ double mutant cells, which show a dramatic reduction in ciliary ARL13B localization, but not in overall cilia frequency, compared with *Mks1*^*krc/krc*^ MEFs. This points to cooperation between IFT and the MKS complex in initiating ciliogenesis and/or in maintaining the cilium.

**Fig 6:**
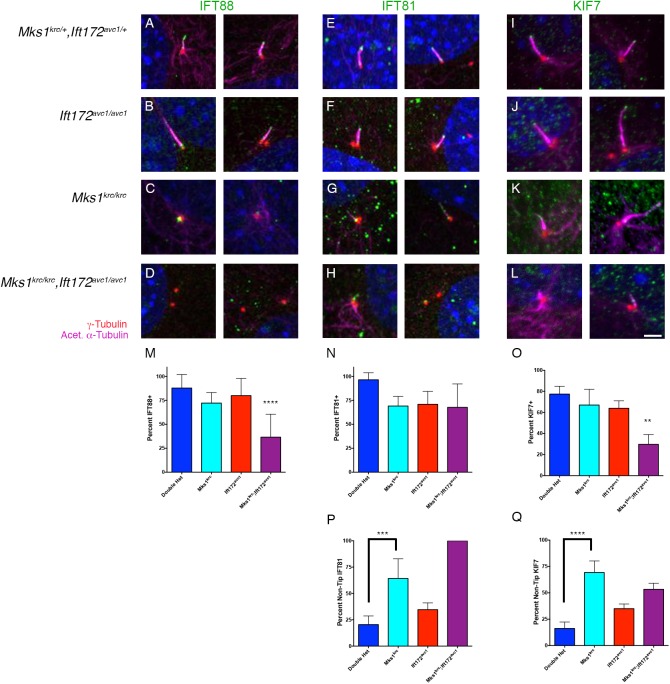
Basal body localization of IFT88 is impaired in *Mks1*^*krc/krc*^*;Ift172*^*avc1/avc1*^ double mutant cells. **(A-L)** MEFs derived from embryos of the indicated genotype were immunostained for IFT88 (A-D, green) IFT81 (E-H, green), or KIF7 (I-L, green) together with γ-Tubulin (red) and acetylated α-Tubulin (magenta) following 48 hours of serum starvation. Two representative images for each condition are shown. Scale bar, 2μm. **(M-O)** Quantitation of the percentage of cells with IFT88 or IFT81 localized to either the basal body or ciliary axoneme. Compared with *Mks1*^*krc/krc*^ or *Ift172*^*avc1/avc1*^ single mutant cells, significantly fewer double mutant cilia were positive for IFT88 localized to the basal body (M, **** p< 0.0001). Localization of IFT81 was not significantly disrupted in double mutants compared with either single mutant, however all of the mutants exhibited reduced localization compared with double heterozygous cells (N, p = 0.0015). The percentage of ciliated cells with KIF7 within the axoneme was also significantly reduced in *Mks1*^*krc/krc*^*;Ift172*^*avc1/avc1*^ double mutants compared with single mutant or double heterozygous cells (O, p< 0.001). Quantification for each genotype was based on imaging of 4 separate fields on 3 coverslips per condition. **(P-Q)** Quantitation of the percentage of cells positive for IFT81 or KIF7 for each genotype in which localization was not restricted to the ciliary tip. Compared with double heterozygous cells, significantly more ciliated *Mks1*^*krc/krc*^ cells exhibited localization of IFT81 (p = 0.0005) or KIF7 (p< 0.0001) that was not restricted to the ciliary tip. Quantification for each genotype was based on imaging of 4 separate fields on 3 coverslips per condition.

To more precisely define the defect in cilium initiation in *Mks1*^*krc/krc*^*; Ift172*^*avc1/avc1*^ mutant cells, we examined the recruitment of IFT proteins by determining the percentage of cells with IFT88 or IFT81 localized to the basal body or ciliary axoneme (Fig 6). For IFT88, we found that the percentage of cells with IFT88 present at the basal body in the double mutant cells (36.5%, n = 76) was significantly lower than the proportion observed in either double heterozygous control MEFs (87.8%, n = 70) or *Mks1*^*krc/krc*^ (72.1%, n = 81) and *Ift172*^*avc1/avc1*^ (79.9%, n = 186) single mutant cells (Fig 6A–6D and 6M). IFT81 was still detected at one of the two centrosomes of *Mks1*^*krc/krc*^*; Ift172*^*avc1/avc1*^ double mutant cells, as well as in the small number of axonemes present in these cells, at similar proportions to both single mutants (Fig 6E–6H and 6N). These results implicate MKS1 and the MKS complex in cooperating with IFT proteins to recruit or retain specific components of the IFT complex at the basal body.

While IFT88 is often seen throughout the ciliary axoneme, as well as concentrating at the ciliary base and tip, IFT81 is typically strongly enriched at the ciliary tip in double heterzygous cells (96.4% of cilia IFT81+, 20.5% with non-tip localized staining, n = 75) [56,59], with localization overlapping that of GLI2 [56]. In *Ift172*^*avc1/avc1*^ cells, localization of IFT81 was slightly reduced relative to double heterozygous cells (70.9%, n = 186), and the percentage of cells with non-tip IFT81 was slightly elevated (34.6%), primarily due to increased localization at the ciliary base in these cells (Fig 6F and 6P). Similar to our observations for GLI2, we found that accumulation of IFT81 at the ciliary tip failed in *Mks1*^*krc/krc*^ mutants (69.1% of cilia IFT81+, 64.2% with non-tip localization, n = 99, Fig 6G and 6P). The same mis-localization of IFT81 was seen in *Mks1*^*krc/krc*^*; Ift172*^*avc1/avc1*^ double mutants, with 100% cilia in these cells (n = 57, 67.7% cilia IFT81+) exhibiting non-restricted localization of IFT81 (Fig 6H and 6P).

IFT81 is a marker for the ciliary tip compartment, which is comprised of the + ends of the axonemal microtubules. The atypical kinesin KIF7 is required for the structure and integrity of the tip compartment [56]. Since the defects in IFT81 ciliary localization are reminiscent of ciliary defects seen in *Kif7* mutant embryos, we examined localization of KIF7 in cells from *Mks1*^*krc/krc*^*; Ift172*^*avc1/avc1*^ mutants as well as both single mutants (Fig 6I and 6L). We found the percentage of ciliated cells with KIF7 present in the axoneme was significantly lower in double mutants compared with other genotypes (Double heterozygous: 77.4% cilia KIF7+, n = 58; *Mks1*^*krc/krc*^: 67.0% cilia KIF7+, n = 59; *Ift172*^*avc1/avc1*^: 63.9% cilia KIF7+, n = 26; Double mutant: 29.7% cilia KIF7+, n = 45;Fig 6O).

The distribution of KIF7 within the axoneme followed a similar pattern to IFT81, consistent with a role for MKS1 and the MKS complex in the cilium tip structure. In WT and *Ift172*^*avc1/avc1*^ mutant cells, KIF7 is predominantly found at the ciliary tip (16.2% and 35% of cells had non-tip localized KIF7, respectively), consistent with previous findings (Fig 6I, 6J and 6Q) [41]. However in *Mks1*^*krc/krc*^ cells, KIF7 was broadly localized throughout the distal axoneme in the majority of cells (69.2% non-tip localized KIF7; Fig 6K and 6Q). The number of double mutant cells with KIF7+ cilia was very small (Fig 6L and 6Q; n = 13), yet KIF7 was still localized in a similar pattern to that exhibited in *Mks1*^*krc/krc*^ mutant cells (53.3% non-tip localized KIF7).

**Fig 7:**
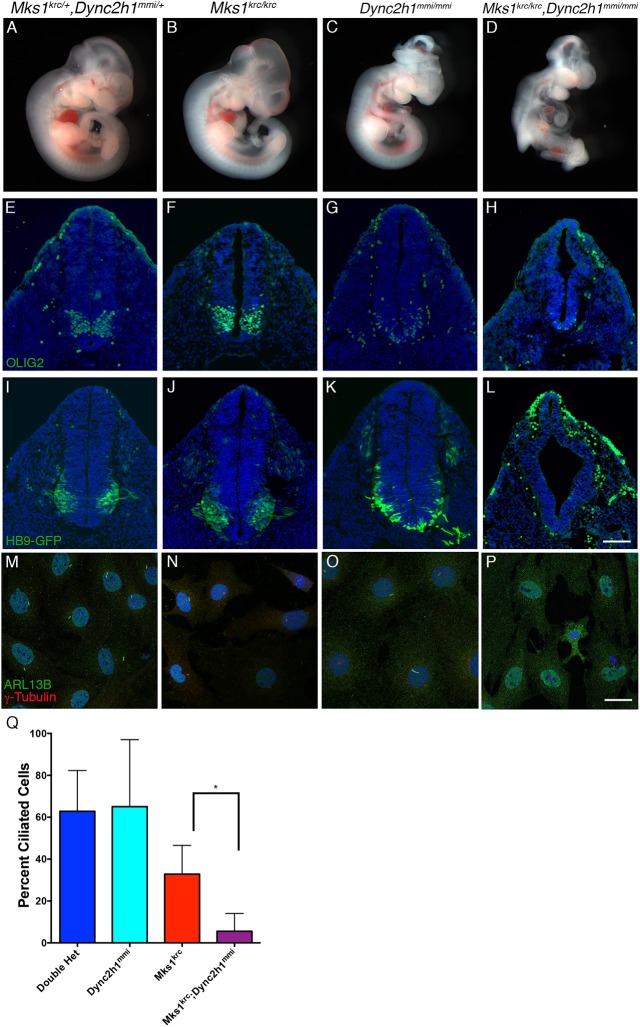
*Mks1*^*krc/krc*^*;Dync2h1*^*mmi/mmi*^ double mutants exhibit enhanced severity of Hh-dependent neural patterning phenotypes, and ciliary trafficking defects. **(A-D)** Double heterozygous (A), *Mks1*^*krc/krc*^ (B), *Dync2h1*^*mmi/mmi*^ (C), or *Mks1*^*krc/krc*^*; Dync2h1*^*mmi/mmi*^ double mutant (D) embryos at E10.5. **(E-L)** Transverse sections through the neural tube of embryos of the indicated genotype at E10.5. Sections were taken at the level of the forelimbs and immunostained with antibodies against OLIG2 (green) to label motor neuron progenitors in E-H. In I-L, embryos expressing an HB9::GFP transgene were examined as an additional marker of motor neuron patterning. While *Dync2h1*^*mmi/mmi*^ single mutants have somewhat reduced numbers of motor neurons (G,K), few or none are present in the *Mks1*^*krc/krc*^*; Dync2h1*^*mmi/mmi*^ double mutants (H,L). **(M-P)** MEFs derived from embryos of each indicated genotype were immunostained for γ-Tubulin (red) and ARL13B (green) to assess cilia formation. Scale bar, 20μm. **(Q)** Quantitation of the percentage of ciliated cells in each genotype positive for either ARL13B following 48 hours of serum starvation. Compared with *Mks1*^*krc/krc*^ single mutant cells, significantly fewer double mutant cells were ciliated (*p = 0.014). Quantification for each genotype was based on imaging of 4 separate fields on 3 coverslips per condition.

We also examined whether *MKS1* interacts genetically with cytoplasmic dynein heavy chain 2 (*Dync2h1*), encoding a component of the dynein motor required for retrograde trafficking in primary cilia [60] (Fig 7). At E10.5, *Mks1*^*krc/krc*^ single mutants appear similar to double heterozygous embryos (Fig 7A and 7B). Similar to other reported *Dync2h1* alleles[5,42], *Dync2h1*^*mmi/mmi*^ embryos frequently exhibit exencephaly at E10.5 (Fig 7C) and die by E11.5 [41]. *Dync2h1*^*mmi/mmi*^*;Mks1*^*krc/krc*^ double mutant embryos exhibit a more severe gross phenotype overall relative to either single mutant, and die by E10.5 (Fig 7D).

To further assess the phenotype of *Dync2h1*^*mmi/mmi*^*;Mks1*^*krc/krc*^ embryos, we examined Hh-dependent neural patterning. *Dync2h1* mutant embryos exhibit severe reductions of the most ventral cell types of the neural tube, but have some motor neurons[5]. For this reason, we examined the specification and patterning of both progenitors labeled by OLIG2 (Fig 7E–7H), and differentiated motor neurons labeled by HB9-GFP (Fig 7I–7L) in *Dync2h1*^*mmi/mmi*^*;Mks1*^*krc/krc*^ embryos as well as each single mutant. As we showed for previous experiments, both motor neuron progenitors and differentiated motor neurons in *Mks1*^*krc/krc*^ single mutant embryos were similar to those of double heterozygotes (Fig 7E, 7F, 7I and 7J). In contrast, *Dync2h1*^*mmi/mmi*^ mutant embryos had reduced numbers of OLIG2+ progenitors (Fig 7G), and differentiated motor neurons span the ventral midline, consistent with the loss of more ventral cell types (Fig 7K). Double mutants exhibit a more severe defect, with a more dramatic reduction in the number of motor neuron progenitors (Fig 7H), and a lack of HB9-GFP+ motor neurons (Fig 7L).

*Dync2h1* mutant embryos have primary cilia, however these cilia are structurally abnormal due to loss of retrograde IFT trafficking [5,42,46]. We next examined cilia formation in cells derived from *Dync2h1*^*mmi/mmi*^*;Mks1*^*krc/krc*^ embryos. Consistent with the neural patterning phenotypes and similar to what we observed for *Mks1*^*krc/krc*^*; Ift172*^*avc1/avc1*^ double mutant embryos, the percentage of ciliated cells was reduced dramatically in *Dync2h1*^*mmi/mmi*^*; Mks1*^*krc/krc*^ double mutant embryos compared with single mutants (Double heterozygous: 62.9% ciliated, n = 102; *Dync2h1*^*mmi/mmi*^: 65.0% ciliated, n = 86; *Mks1*^*krc/krc*^: 32.9% ciliated, n = 106; double mutant: 5.5% ciliated, n = 104; Fig 7M–7Q, S3 Fig). These data further support a role for MKS1 and the MKS complex in promoting IFT.

In addition to genetically testing whether MKS1 cooperates with the IFT complex to promote ciliary trafficking, we also examined whether a component of the BBSome genetically interacts with IFT172 by generating *Bbs4*^*-/-*^*; Ift172*^*avc1/avc1*^ double mutants. BBSome proteins have been previously implicated in IFT: BBSome proteins traffic through in cilia in *Chlamydomonas* as IFT cargo [61], and BBS7 and 8 are required for normal IFT in *C*. *elegans* [62]. However, in contrast to our findings with *Mks1*^*krc/krc*^*; Ift172*^*avc1/avc1*^ double mutant embryos, we did not observe any enhancement of phenotype of these double mutants, relative to *Ift172*^*avc1/avc1*^ single mutants (S5 Fig).

## Discussion

**Fig 8:**
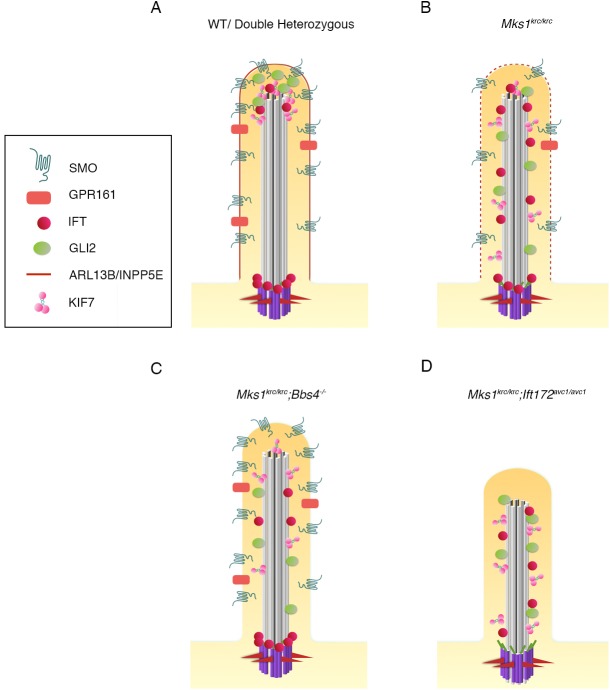
Model summarizing the trafficking defects in *Mks1*^*krc/krc*^ mutants as well as double mutants with *Bbs* and *Ift*. All conditions are depicted in the state of HH pathway activation upon stimulation with SAG. **(A)** WT and double heterozygous cells. Red outline depicts the normal ciliary membrane integrity (based on ARL13b and INPP5E localization) SMO is present in the ciliary membrane, GPR161 is reduced within the ciliary membrane due to pathway activation. GLI2 and KIF7 are enriched at the ciliary tip. IFT components are enriched at the ciliary tip and the transition zone near the basal body (shown in purple). **(B)** *Mks1*^*krc/krc*^ cells exhibit defects in the localization of ciliary membranes (dashed red line)- ARL13b is reduced and INPP5E is absent. SMO is reduced, and GLI2, KIF7, and IFT81 are no longer restricted to the ciliary tip. **(C)** *Mks1*^*krc/krc*^ cells that also lack BBS4 exhibit additional defects in the trafficking of ciliary membrane proteins. ARL13B is absent, however SMO is restored in the cilia by the loss of BBS4. GLI2 is not restricted to the ciliary tip, as with *Mks1*^*krc/krc*^ single mutant cells, however a lower percentage of cells have ciliary GLI2. **(D)** *Mks1*^*krc/krc*^ cells that also have impaired IFT function due to a homozygous hypomorphic mutation in *Ift172* have severe ciliogenesis defects. Less than 10% of *Mks1*^*krc/krc*^*;Ift172*^*avc1/avc1*^ cells are ciliated. Those cilia that are present are shorter than normal. IFT88 is absent from the transition zone or the cilium, while IFT81 and KIF7 are no longer restricted to the tip, as with *Mks1*^*krc/krc*^ single mutant cells.

In this study, we show genetic interactions between MKS1 and the BBSome component BBS4, as well as between MKS1 and IFT machinery. Our data suggest that the BBSome and MKS complex components work both together and also have opposing roles in regulating the trafficking of proteins to the ciliary membrane. We show that BBSome and MKS complex components cooperate in targeting of ARL13B to the ciliary membrane, but have opposing effects on SMO localization, and to a lesser extent GPR161 in response to Hh pathway signaling: loss of *Bbs4* on and *Mks1* mutant background partially restores SMO and GPR161 localization. Another membrane protein, INPP5E, depends primarily on MKS1 for localization to the cilium, similar to results reported for other components of the MKS complex [33].These varied membrane trafficking defects in the double mutants point to these cells having a disrupted complement of ciliary membrane proteins, which is likely an important contributor to the exacerbated defects observed in the *Bbs4*^*-/-*^*; Mks1*^*krc/krc*^ double mutant embryos (Summarized in Fig 8A–8C, and Table 1).

Our data are in broad agreement with recent work showing genetic interactions between other components of the MKS complex and the BBSome [33], however differences in our findings also uncover some important additional aspects of how these two complexes function together in ciliogenesis. For example, Yee *et al*. show that double mutants between *Bbs1* and the MKS complex component *Tctn1* form cilia at a substantially lower frequency than *Tctn1*^*-/-*^ single mutants [33]. In contrast, our *Bbs4*^*-/-*^*; Mks1*^*krc/krc*^ mutants form acetylated α-Tubulin positive cilia at nearly the same frequency as *Mks1*^*krc/krc*^ single mutants, however the cilia in the double mutants lack ARL13B. This difference is possibly due to the specific alleles used. For example, evidence suggests that BBS1 may play a more essential role in the stability of the BBSome complex than does BBS4 [63]. Nevertheless, perhaps by perturbing BBSome function more weakly, our data reveal that the MKS complex and BBSome cooperate to mediate trafficking of specific membrane proteins, such as ARL13B, to the cilium. Our data showing partial restoration of trafficking of some membrane proteins, such as SMO and GPR161, but not others upon impairment of BBSome function indicates a complex relationship between the MKS proteins and BBSome in ciliary membrane trafficking.

We identify strong genetic interactions between *Mks1* and components of the IFT machinery: in double mutants between *Mks1* and either a hypomorphic allele of *Ift172*, or *Dync2h1*^*mmi*^ we observe not only a reduction in ciliary protein trafficking, but also a failure of cilium assembly. This suggests that in addition to the previously described roles for the MKS complex in mediating entry of specific membrane proteins into cilia, MKS1 and the MKS complex cooperate with IFT to mediate the assembly of the axoneme. While the precise mechanisms by which MKS1 mediates IFT remain to be defined, localization or retention of IFT88 to the mother centriole is disrupted in *Mks1*^*krc/krc*^*; Ift172*^*avc1/avc1*^ mutant cells, suggesting that MKS transition zone complex proteins may cooperate with IFT proteins to promote the recruitment or stable retention of IFT complexes at the basal body. This in turn contributes defects in cilium initiation in double mutant cells, and results in the dramatic exacerbation of Hh-related patterning defects seen in these embryos (For summary see Fig 8D, Table 1).

We note that recruitment of IFT88 to the basal body was disrupted by the combined impairment of MKS1 and IFT172, while IFT81 recruitment in the double mutants did not differ from either of the single mutants (Fig 6). A biochemical study of IFT-B proteins found that IFT81 and IFT88 are components of separate IFTB sub-complexes, bridged by a shared interaction with another IFT-B protein, IFT52 [64]. This could explain why these two IFT-B particle components are differentially affected in the *Mks1*^*krc/krc*^*; Ift172*^*avc1/avc1*^ mutant cells.

MKS1 and the MKS complex may also directly or indirectly affect IFT trafficking, and/or the structure of the ciliary tip compartment. For example, we find that GLI2, IFT81 and KIF7 localization in *Mks1*^*krc/krc*^ single mutants is abnormal, with these proteins failing to enrich at the ciliary tip, and instead becoming localized to either a broader portion of the distal axoneme, or diffused throughout the entire axoneme, suggesting that MKS1 may be important for the structure of the ciliary tip. *Mks1*^*krc/krc*^*; Ift172*^*avc1/avc1*^ double mutants also exhibit defects in IFT81 and KIF7 localization as well as a general reduction in the proportion of cilia that are positive for KIF7. Thus, our model is that MKS proteins at the transition zone cooperate with IFT to mediate cilium initiation, through recruiting or maintaining a subset of IFT proteins at the basal body (for example, IFT88). In double mutants, those cilia that are present also have defects in ciliary trafficking and structure, due to a direct or indirect role for MKS1 in the structure of the cilia tip compartment. The double mutants also reveal a cooperative role for MKS and IFT in recruitment of KIF7, a kinesin important for axoneme structure, to the cilium.

A connection between a transition zone complex and IFT was also recently found in *C*. *elegans* through the genetic interaction of *nphp4*, which encodes a component of the NPHP transition zone complex, and *osm-3* [22]. *Osm3* encodes a kinesin important for IFT in the distal segment of the cilium [65], the vertebrate homolog of which is known as Kif17 [66]. *Osm-3* did not genetically interact with MKS complex components in this study, however another study revealed that some of the functions of the MKS and NPHP transition zone complexes may have shifted in the course of evolution [33].

Our data on the relationship between the BBSome component BBS4 and IFT contrasts with another report, which showed that double mutants between *Bbs7* and a hypomorphic allele of *Ift88* (*Ift88*^*orpk*^) exhibited enhanced Hh-related patterning defects and embryonic lethality [55]. The reason for this difference in findings could be due to differences in the roles of the distinct protein components of the BBSome with respect to IFT function. For example, BBS7 is unique in that, in addition its role as a core component of the BBSome, it also physically interacts with the BBS chaperonin complex [67], a group of BBS-associated proteins that help to assemble the BBSome [68]. The synergistic phenotypic interaction between *Bbs7* and *Ift88* could therefore be due to this additional role of BBS7.

Taken together, our findings and those of others show that the separate protein complexes important for cilia structure and trafficking cooperate together to mediate specific aspects of cilia formation. The differences between our findings and those of other recent works highlight the fact that the distinct protein components of these complexes also have unique roles within the complex and could therefore have different relationships with other ciliary protein modules. Further exploration of this area will continue to reveal new information about how ciliogenesis is regulated as well as enhancing our understanding of the genetic complexity seen in ciliopathies.

## Supporting information

S1 Fig MKS complex component TMEM67 is lost from the transition zone of *Mks1*^*krc/krc*^ mutant cells. **(A)** Mks1^krc/+^ cells have TMEM67 (green), another component of the MKS protein complex, localized to the transition zone between the centrosome (red) and cilium (magenta). Arrowheads indicate TMEM67 at the transition zone.
**(B)** TMEM67 localization is absent from the transition zone in Mks1^krc/krc^ mutant cells. Scale bar, 5μm.
(TIF)

S2 Fig Gli1 expression levels in Mks1-Bbs4 and Mks1-Ift172 mutant cells. qPCR comparing expression levels of *Gli1* in response to stimulation of cells with SAG for *Mks1*^*krc/krc*^*;Bbs4*^*-/-*^ and *Mks1*^*krc/krc*^*;Ift172*^*avc1/avc1*^ double mutant cells as well as WT and single mutant cells. Expression was analyzed with three biological replicates for each experimental condition, with three technical replicates for each biological replicate. The graphs depict the average relative *Gli2* levels (normalized to GAPDH for each replicate), error bars are standard deviation.
(TIF)

S3 Fig SEM images of cilia from mutant embryos. **(A-H)** Embryos of the indicated genotypes were collected at E10.5, and the neural tubes were dissected out and fixed. Neural tubes were then opened and imaged *en face* by SEM. The regions imaged correspond to the ventral portion of the neural tube, anterior to the forelimbs. Scale bar, 1μm.
**(I)** Quantification of the percentage of cells that are ciliated within the neural tube for each genotype. E10.5 SEM images of the neural tube were imaged as in A-H. A minimum of 2 fields were imaged for each of 3 embryos for each genotype at the forelimb level. In each field, cells with clear boundaries were counted and scored for whether or not they had a cilium. The percentage of ciliated cells for each field was recorded. Bars represent the mean of all fields examined for each genotype, and error bars are standard deviation. Genotypes were compared using ANOVA with a Tukey-Kramer multiple-comparison correction. Similar to our data based on percentage of ciliated cells in MEFs: we find that cilia frequency in *Bbs4*^*-/-*^, *Ift172*^*avc1/avc1*^, and *Dync2h1*^*mmi/mmi*^ single mutants is comparable to WT embryos. *Mks1*^*krc/krc*^*;Bbs4*^*-/-*^ have similar numbers of cilia to *Mks1*^*krc/krc*^ single mutants (p>0.99), however both *Mks1*^*krc/krc*^*; Dync2h1*^*mmi/mmi*^ and *Mks1*^*krc/krc*^*;Ift172*^*avc1/avc1*^ double mutants have significantly fewer cilia than *Mks1*^*krc/krc*^ single mutants (p<0.0001, and p = 0.0002, respectively).
(TIF)

S4 Fig Quantification of membrane protein localization. Related to Fig 3. **(A-D)** Each graph shows the percentage of cilia positive for the indicated marker. Bars represent the mean percent positive cilia for each genotype, error bars represent standard deviation.
**(E)** The graph represents the percentage of GLI2+ cilia for each genotype (upon SAG treatment) in which GLI2 was not restricted to the ciliary tip (extending more than 1/3 of the length of the cilium from the tip, or seen in a location of the cilium other than the tip such as the base). Bars represent the mean percent of GLI2+ cilia with non-tip GLI2, error bars represent standard deviation.
(TIF)

S5 Fig *Bbs4*^*-/-*^*;Ift172*^*avc1/avc1*^ double mutant embryos do not have enhanced Shh-related phenotypes compared with individual mutants. **(A-D)** Double heterozygous (A), *Bbs4*^*-/-*^ (B), *Ift172*^*avc1/avc1*^ (C), or *Ift172*^*avc1/avc1*^*;Bbs4*^*-/-*^ double mutant (D) embryos at E10.5.
**(E-H)** Transverse sections through the neural tube of embryos of the indicated genotype at E10.5. Sections were taken at the level of the forelimbs and immunostained with antibodies against FOXA2 (red) and ISL1 (green). Scale bar, 100μm.
(TIF)

S1 Table Total numbers of embryos recovered for each genotype in double mutant crosses. (DOCX)

## Abbreviations

IFT: Intraflagellar transport
GPCR: G Protein coupled receptor
MKS: Meckel’s syndrome
BBS: Bardet Biedl syndrome
Hh: Hedgehog
Shh: Sonic hedgehog
MEF: Mouse embryonic fibroblast

## References

[1] Badano JL, Mitsuma N, Beales PL, Katsanis N. The ciliopathies: an emerging class of human genetic disorders. Annual review of genomics and human genetics. 2006;7: 125–148. 10.1146/annurev.genom.7.080505.115610 16722803

[2] Baker K, Beales PL. Making sense of cilia in disease: The human ciliopathies. Parisi MA, Toriello HV, editors. Am J Med Genet. 2009;151C: 281–295. 10.1002/ajmg.c.30231 19876933

[3] Goetz SC, Anderson KV. The primary cilium: a signalling centre during vertebrate development. Nat Rev Genet. 2010;11: 331–344. 10.1038/nrg2774 20395968 PMC3121168

[4] Huangfu D, Liu A, Rakeman AS, Murcia NS, Niswander L, Anderson KV. Hedgehog signalling in the mouse requires intraflagellar transport proteins. Nature. 2003;426: 83–87. 10.1038/nature02061 14603322

[5] Huangfu D, Anderson KV. Cilia and Hedgehog responsiveness in the mouse. Proc Natl Acad Sci USA. National Acad Sciences; 2005;102: 11325–11330. 10.1073/pnas.0505328102 16061793 PMC1183606

[6] Liu A, Wang B, Niswander LA. Mouse intraflagellar transport proteins regulate both the activator and repressor functions of Gli transcription factors. Development. 2005;132: 3103–3111. 10.1242/dev.01894 15930098

[7] Tran PV, Haycraft CJ, Besschetnova TY, Turbe-Doan A, Stottmann RW, Herron BJ, et al THM1 negatively modulates mouse sonic hedgehog signal transduction and affects retrograde intraflagellar transport in cilia. Nat Genet. 2008;40: 403–410. 10.1038/ng.105 18327258 PMC4817720

[8] Berbari NF, Lewis JS, Bishop GA, Askwith CC, Mykytyn K. Bardet-Biedl syndrome proteins are required for the localization of G protein-coupled receptors to primary cilia. Proc Natl Acad Sci USA. 2008;105: 4242–4246. 10.1073/pnas.0711027105 18334641 PMC2393805

[9] Domire JS, Green JA, Lee KG, Johnson AD, Askwith CC, Mykytyn K. Dopamine receptor 1 localizes to neuronal cilia in a dynamic process that requires the Bardet-Biedl syndrome proteins. Cell Mol Life Sci. 2011;68: 2951–2960. 10.1007/s00018-010-0603-4 21152952 PMC3368249

[10] Green JA, Mykytyn K. Neuronal ciliary signaling in homeostasis and disease. Cell Mol Life Sci. 2010;67: 3287–3297. 10.1007/s00018-010-0425-4 20544253 PMC3349968

[11] Gerdes JM, Davis EE, Katsanis N. The Vertebrate Primary Cilium in Development, Homeostasis, and Disease. Cell. 2009;137: 32–45. 10.1016/j.cell.2009.03.023 19345185 PMC3016012

[12] Alvarez Retuerto AI, Cantor RM, Gleeson JG, Ustaszewska A, Schackwitz WS, Pennacchio LA, et al Association of common variants in the Joubert syndrome gene (AHI1) with autism. Hum Mol Genet. Oxford University Press; 2008;17: 3887–3896. 10.1093/hmg/ddn291 18782849 PMC2638573

[13] Migliavacca E, Golzio C, Männik K, Blumenthal I, Oh EC, Harewood L, et al A Potential Contributory Role for Ciliary Dysfunction in the 16p11.2 600 kb BP4-BP5 Pathology. Am J Hum Genet. 2015;96: 784–796. 10.1016/j.ajhg.2015.04.002 25937446 PMC4570289

[14] Marley A, Zastrow von M. A Simple Cell-Based Assay Reveals That Diverse Neuropsychiatric Risk Genes Converge on Primary Cilia. PLoS ONE. Public Library of Science; 2012;7: e46647 10.1371/journal.pone.0046647 23056384 PMC3463515

[15] Doherty D. Joubert syndrome: insights into brain development, cilium biology, and complex disease. Semin Pediatr Neurol. 2009;16: 143–154. 10.1016/j.spen.2009.06.002 19778711 PMC2804071

[16] Leitch CC, Zaghloul NA, Davis EE, Stoetzel C, Diaz-Font A, Rix S, et al Hypomorphic mutations in syndromic encephalocele genes are associated with Bardet-Biedl syndrome. Nat Genet. 2008;40: 443–448. 10.1038/ng.97 18327255

[17] Karmous-Benailly H, Martinovic J, Gubler M-C, Sirot Y, Clech L, Ozilou C, et al Antenatal presentation of Bardet-Biedl syndrome may mimic Meckel syndrome. Am J Hum Genet. 2005;76: 493–504. 10.1086/428679 15666242 PMC1196400

[18] Castro-Sánchez S, Álvarez-Satta M, Cortón M, Guillén E, Ayuso C, Valverde D. Exploring genotype-phenotype relationships in Bardet-Biedl syndrome families. J Med Genet. BMJ Publishing Group Ltd; 2015;52: 503–513. 10.1136/jmedgenet-2015-103099 26082521

[19] Beales PL, Badano JL, Ross AJ, Ansley SJ, Hoskins BE, Kirsten B, et al Genetic interaction of BBS1 mutations with alleles at other BBS loci can result in non-Mendelian Bardet-Biedl syndrome. Am J Hum Genet. 2003;72: 1187–1199. 10.1086/375178 12677556 PMC1180271

[20] Hoefele J, Wolf MTF, O'Toole JF, Otto EA, Schultheiss U, Dêschenes G, et al Evidence of oligogenic inheritance in nephronophthisis. Journal of the American Society of Nephrology. American Society of Nephrology; 2007;18: 2789–2795. 10.1681/ASN.2007020243 17855640

[21] Katsanis N, Ansley SJ, Badano JL, Eichers ER, Lewis RA, Hoskins BE, et al Triallelic Inheritance in Bardet-Biedl Syndrome, a Mendelian Recessive Disorder. Science. American Association for the Advancement of Science; 2001;293: 2256–2259. 10.1126/science.1063525 11567139

[22] Masyukova SV, Landis DE, Henke SJ, Williams CL, Pieczynski JN, Roszczynialski KN, et al A Screen for Modifiers of Cilia Phenotypes Reveals Novel MKS Alleles and Uncovers a Specific Genetic Interaction between osm-3 and nphp-4. Beier DR, editor. PLoS Genet. Public Library of Science; 2016;12: e1005841 10.1371/journal.pgen.1005841 26863025 PMC4749664

[23] Davis EE, Katsanis N. The ciliopathies: a transitional model into systems biology of human genetic disease. Current Opinion in Genetics & Development. 2012;22: 290–303. 22632799 10.1016/j.gde.2012.04.006 PMC3509787

[24] Davis EE, Zhang Q, Liu Q, Diplas BH, Davey LM, Hartley J, et al TTC21B contributes both causal and modifying alleles across the ciliopathy spectrum. Nat Genet. 2011;43: 189–196. 10.1038/ng.756 21258341 PMC3071301

[25] Pedersen LB, Veland IR, Schrøder JM, Christensen ST. Assembly of primary cilia. Dev Dyn. 2008;237: 1993–2006. 10.1002/dvdy.21521 18393310

[26] Bialas NJ, Inglis PN, Li C, Robinson JF, Parker JDK, Healey MP, et al Functional interactions between the ciliopathy-associated Meckel syndrome 1 (MKS1) protein and two novel MKS1-related (MKSR) proteins. J Cell Sci. 2009;122: 611–624. 10.1242/jcs.028621 19208769 PMC2720918

[27] Dawe HR, Smith UM, Cullinane AR, Gerrelli D, Cox P, Badano JL, et al The Meckel-Gruber Syndrome proteins MKS1 and meckelin interact and are required for primary cilium formation. Hum Mol Genet. 2007;16: 173–186. 10.1093/hmg/ddl459 17185389

[28] Nachury MV, Loktev AV, Zhang Q, Westlake CJ, Peranen J, Merdes A, et al A core complex of BBS proteins cooperates with the GTPase Rab8 to promote ciliary membrane biogenesis. Cell. 2007;129: 1201–1213. 10.1016/j.cell.2007.03.053 17574030

[29] Jin H, White SR, Shida T, Schulz S, Aguiar M, Gygi SP, et al The Conserved Bardet-Biedl Syndrome Proteins Assemble a Coat that Traffics Membrane Proteins to Cilia. Cell. 2010;141: 1208–1219. 10.1016/j.cell.2010.05.015 20603001 PMC2898735

[30] Taschner M, Bhogaraju S, Lorentzen E. Architecture and function of IFT complex proteins in ciliogenesis. Differentiation. 2012;83: S12–S22. 10.1016/j.diff.2011.11.001 22118932 PMC3977345

[31] Garcia-Gonzalo FR, Corbit KC, Sirerol-Piquer MS, Ramaswami G, Otto EA, Noriega TR, et al A transition zone complex regulates mammalian ciliogenesis and ciliary membrane composition. Nat Genet. 2011;43: 776–784. 10.1038/ng.891 21725307 PMC3145011

[32] Chih B, Liu P, Chinn Y, Chalouni C, Komuves LG, Hass PE, et al A ciliopathy complex at the transition zone protects the cilia as a privileged membrane domain. Nat Cell Biol. 2011;14: 61–72. 10.1038/ncb2410 22179047

[33] Yee LE, Garcia-Gonzalo FR, Bowie RV, Li C, Kennedy JK, Ashrafi K, et al Conserved Genetic Interactions between Ciliopathy Complexes Cooperatively Support Ciliogenesis and Ciliary Signaling. PLoS Genet. 2015;11: e1005627 10.1371/journal.pgen.1005627 26540106 PMC4635004

[34] Huber C, Cormier-Daire V. Ciliary disorder of the skeleton. Unger S, Bonafé L, Superti-Furga A, editors. Am J Med Genet. 2012;160C: 165–174. 10.1002/ajmg.c.31336 22791528

[35] Perrault I, Halbritter J, Porath JD, Gérard X, Braun DA, Gee HY, et al IFT81, encoding an IFT-B core protein, as a very rare cause of a ciliopathy phenotype. J Med Genet. 2015;52: 657–665. 10.1136/jmedgenet-2014-102838 26275418 PMC4621372

[36] Beales PL, Bland E, Tobin JL, Bacchelli C, Tuysuz B, Hill J, et al IFT80, which encodes a conserved intraflagellar transport protein, is mutated in Jeune asphyxiating thoracic dystrophy. Nat Genet. 2007;39: 727–729. 10.1038/ng2038 17468754

[37] Halbritter J, Bizet AA, Schmidts M, Porath JD, Braun DA, Gee HY, et al Defects in the IFT-B component IFT172 cause Jeune and Mainzer-Saldino syndromes in humans. Am J Hum Genet. 2013;93: 915–925. 10.1016/j.ajhg.2013.09.012 24140113 PMC3824130

[38] Kulaga HM, Leitch CC, Eichers ER, Badano JL, Lesemann A, Hoskins BE, et al Loss of BBS proteins causes anosmia in humans and defects in olfactory cilia structure and function in the mouse. Nat Genet. 2004;36: 994–998. 10.1038/ng1418 15322545

[39] Weatherbee SD, Niswander LA, Anderson KV. A mouse model for Meckel syndrome reveals Mks1 is required for ciliogenesis and Hedgehog signaling. Hum Mol Genet. Oxford University Press; 2009;18: 4565–4575. 10.1093/hmg/ddp422 19776033 PMC2773271

[40] Friedland-Little JM, Hoffmann AD, Ocbina PJR, Peterson MA, Bosman JD, Chen Y, et al A novel murine allele of Intraflagellar Transport Protein 172 causes a syndrome including VACTERL-like features with hydrocephalus. Hum Mol Genet. 2011;20: 3725–3737. 10.1093/hmg/ddr241 21653639 PMC3168284

[41] Liem KF, He M, Ocbina PJR, Anderson KV. Mouse Kif7/Costal2 is a cilia-associated protein that regulates Sonic hedgehog signaling. Proc Natl Acad Sci USA. 2009;106: 13377–13382. 10.1073/pnas.0906944106 19666503 PMC2726420

[42] Ocbina PJR, Anderson KV. Intraflagellar transport, cilia, and mammalian Hedgehog signaling: analysis in mouse embryonic fibroblasts. Dev Dyn. 2008;237: 2030–2038. 10.1002/dvdy.21551 18488998 PMC2702862

[43] Rohatgi R, Milenkovic L, Scott MP. Patched1 Regulates Hedgehog Signaling at the Primary Cilium. Science. 2007;317: 372–376. 10.1126/science.1139740 17641202

[44] Cho A, Ko HW, Eggenschwiler JT. FKBP8 cell-autonomously controls neural tube patterning through a Gli2- and Kif3a-dependent mechanism. Dev Biol. 2008;321: 27–39. 10.1016/j.ydbio.2008.05.558 18590716

[45] Caspary T, Larkins CE, Anderson KV. The Graded Response to Sonic Hedgehog Depends on Cilia Architecture. Dev Cell. 2007;12: 767–778. 10.1016/j.devcel.2007.03.004 17488627

[46] Ocbina PJR, Eggenschwiler JT, Moskowitz I, Anderson KV. Complex interactions between genes controlling trafficking in primary cilia. Nat Genet. 2011;43: 547–553. 10.1038/ng.832 21552265 PMC3132150

[47] Chávez M, Ena S, Van Sande J, de Kerchove d'Exaerde A, Schurmans S, Schiffmann SN. Modulation of Ciliary Phosphoinositide Content Regulates Trafficking and Sonic Hedgehog Signaling Output. Dev Cell. 2015;34: 338–350. 10.1016/j.devcel.2015.06.016 26190144

[48] Cui C, Chatterjee B, Francis D, Yu Q, Sanagustin JT, Francis R, et al Disruption of Mks1 localization to the mother centriole causes cilia defects and developmental malformations in Meckel-Gruber syndrome. Disease Models & Mechanisms. 2010;: 1–14. 10.1242/dmm.006262 PMC3008963 21045211

[49] Mykytyn K, Mullins RF, Andrews M, Chiang AP, Swiderski RE, Yang B, et al Bardet-Biedl syndrome type 4 (BBS4)-null mice implicate Bbs4 in flagella formation but not global cilia assembly. Proc Natl Acad Sci USA. 2004;101: 8664–8669. 10.1073/pnas.0402354101 15173597 PMC423252

[50] Bielas SL, Silhavy JL, Brancati F, Kisseleva MV, Al-Gazali L, Sztriha L, et al Mutations in INPP5E, encoding inositol polyphosphate-5-phosphatase E, link phosphatidyl inositol signaling to the ciliopathies. Nat Genet. 2009;41: 1032–1036. 10.1038/ng.423 19668216 PMC2746682

[51] Jacoby M, Cox JJ, Gayral S, Hampshire DJ, Ayub M, Blockmans M, et al INPP5E mutations cause primary cilium signaling defects, ciliary instability and ciliopathies in human and mouse. Nat Genet. 2009;41: 1027–1031. 10.1038/ng.427 19668215

[52] Mukhopadhyay S, Wen X, Ratti N, Loktev A, Rangell L, Scales SJ, et al The Ciliary G-Protein-Coupled Receptor Gpr161 Negatively Regulates the Sonic Hedgehog Pathway via cAMP Signaling. Dev Cell. 2013;152: 210–223. 10.1016/j.cell.2012.12.026 23332756

[53] Humbert MC, Weihbrecht K, Searby CC, Li Y, Pope RM, Sheffield VC, et al ARL13B, PDE6D, and CEP164 form a functional network for INPP5E ciliary targeting. Proc Natl Acad Sci USA. National Acad Sciences; 2012;109: 19691–19696. 10.1073/pnas.1210916109 23150559 PMC3511769

[54] Corbit KC, Aanstad P, Singla V, Norman AR, Stainier DYR, Reiter JF. Vertebrate Smoothened functions at the primary cilium. Nature. 2005;437: 1018–1021. 10.1038/nature04117 16136078

[55] Zhang Q, Seo S, Bugge K, Stone EM, Sheffield VC. BBS proteins interact genetically with the IFT pathway to influence SHH-related phenotypes. Hum Mol Genet. 2012;21: 1945–1953. 10.1093/hmg/dds004 22228099 PMC3315203

[56] He M, Subramanian R, Bangs F, Omelchenko T, Liem KF, Kapoor TM, et al The kinesin-4 protein Kif7 regulates mammalian Hedgehog signalling by organizing the cilium tip compartment. Nat Cell Biol. 2014;16: 663–672. 10.1038/ncb2988 24952464 PMC4085576

[57] Goetz SC, Liem KF, Anderson KV. The spinocerebellar ataxia-associated gene tau tubulin kinase 2 controls the initiation of ciliogenesis. Cell. 2012;151: 847–858. 10.1016/j.cell.2012.10.010 23141541 PMC3496184

[58] Marszalek JR, Ruiz-Lozano P, Roberts E, Chien KR, Goldstein LS. Situs inversus and embryonic ciliary morphogenesis defects in mouse mutants lacking the KIF3A subunit of kinesin-II. Proc Natl Acad Sci USA. 1999;96: 5043–5048. 10220415 10.1073/pnas.96.9.5043 PMC21813

[59] Bhogaraju S, Cajánek L, Fort C, Blisnick T, Weber K, Taschner M, et al Molecular Basis of Tubulin Transport Within the Cilium by IFT74 and IFT81. Science. American Association for the Advancement of Science; 2013;341: 1009–1012. 10.1126/science.1240985 23990561 PMC4359902

[60] Scholey JM. Intraflagellar transport motors in cilia: moving along the cell's antenna. J Cell Biol. 2008;180: 23–29. 10.1083/jcb.200709133 18180368 PMC2213603

[61] Lechtreck K-F, Johnson EC, Sakai T, Cochran D, Ballif BA, Rush J, et al The Chlamydomonas reinhardtii BBSome is an IFT cargo required for export of specific signaling proteins from flagella. J Cell Biol. 2009;187: 1117–1132. 10.1083/jcb.200909183 20038682 PMC2806276

[62] Blacque OE. Loss of C. elegans BBS-7 and BBS-8 protein function results in cilia defects and compromised intraflagellar transport. Genes & Development. 2004;18: 1630–1642. 15231740 10.1101/gad.1194004 PMC443524

[63] Seo S, Zhang Q, Bugge K, Breslow DK, Searby CC, Nachury MV, et al A Novel Protein LZTFL1 Regulates Ciliary Trafficking of the BBSome and Smoothened. PLoS Genet. Public Library of Science; 2011;7: e1002358 10.1371/journal.pgen.1002358 22072986 PMC3207910

[64] Taschner M, Kotsis F, Braeuer P, Kuehn EW, Lorentzen E. Crystal structures of IFT70/52 and IFT52/46 provide insight into intraflagellar transport B core complex assembly. J Cell Biol. Rockefeller Univ Press; 2014;207: 269–282. 10.1083/jcb.201408002 25349261 PMC4210449

[65] Shakir MA, Fukushige T, Yasuda H, Miwa J, Siddiqui SS. C. elegans osm-3 gene mediating osmotic avoidance behaviour encodes a kinesin-like protein. NeuroReport. 1993;4: 891–894. 7690265 10.1097/00001756-199307000-00013

[66] Insinna C, Pathak N, Perkins B, Drummond I, Besharse JC. The homodimeric kinesin, Kif17, is essential for vertebrate photoreceptor sensory outer segment development. Dev Biol. 2008;316: 160–170. 10.1016/j.ydbio.2008.01.025 18304522 PMC2362383

[67] Zhang Q, Nishimura D, Vogel T, Shao J, Swiderski R, Yin T, et al BBS7 is required for BBSome formation and its absence in mice results in Bardet-Biedl syndrome phenotypes and selective abnormalities in membrane protein trafficking. J Cell Sci. The Company of Biologists Ltd; 2013;126: 2372–2380. 10.1242/jcs.111740 23572516 PMC3679484

[68] Seo S, Baye LM, Schulz NP, Beck JS, Zhang Q, Slusarski DC, et al BBS6, BBS10, and BBS12 form a complex with CCT/TRiC family chaperonins and mediate BBSome assembly. Proc Natl Acad Sci USA. National Acad Sciences; 2010;107: 1488–1493. 10.1073/pnas.0910268107 20080638 PMC2824390

