# Supplementary figures and images for: The Meckel syndrome- associated protein MKS1 functionally interacts with components of the BBSome and IFT complexes to mediate ciliary trafficking and hedgehog signaling
Source: PLoS One. 2017 Mar 14;12(3):e0173399. doi: 10.1371/journal.pone.0173399 (PMC5349470; doi:10.1371/journal.pone.0173399)

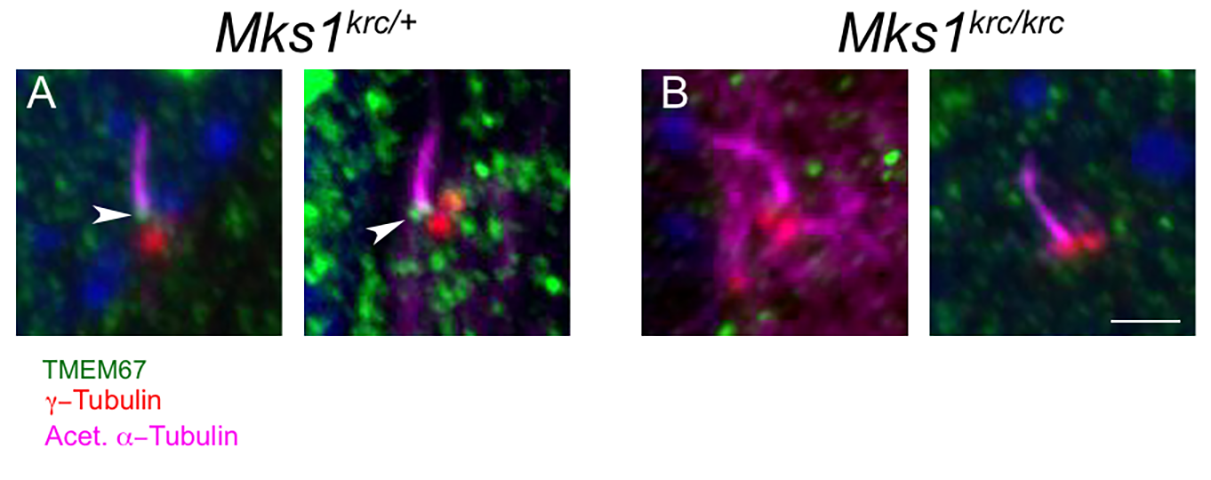

Supplement: S1 Fig — (A) Mks1krc/+ cells have TMEM67 (green), another component of the MKS protein complex, localized to the transition zone between the centrosome (red) and cilium (magenta). Arrowheads indicate TMEM67 at the transition zone. (B) TMEM67 localization is absent from the transition zone in Mks1krc/krc mutant cells. Scale bar, 5μm. (TIF) [file pone.0173399.s001.tif]

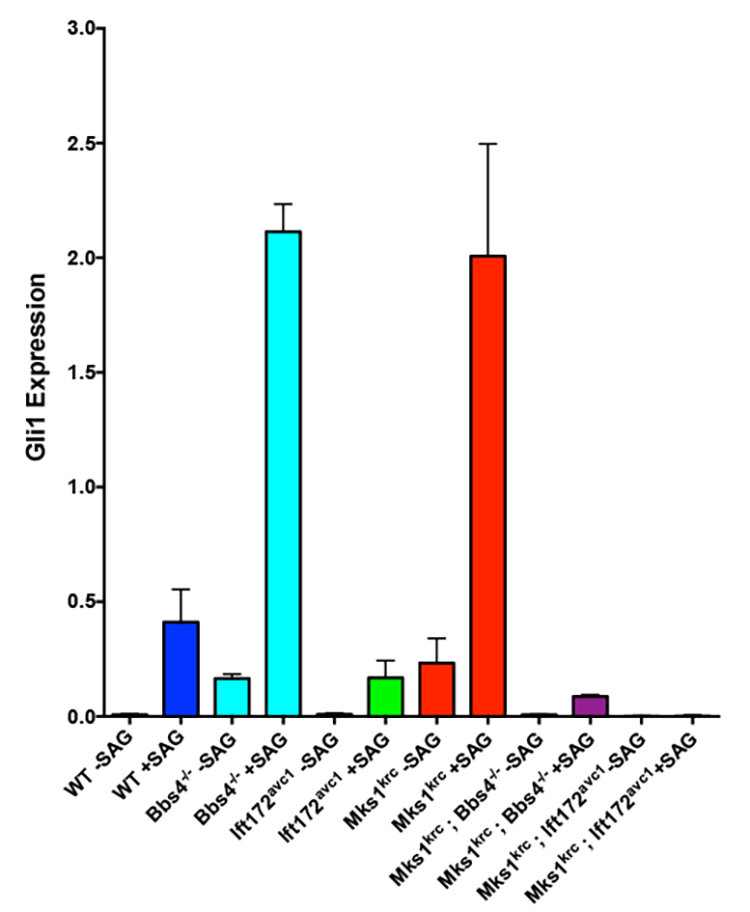

Supplement: S2 Fig — qPCR comparing expression levels of Gli1 in response to stimulation of cells with SAG for Mks1krc/krc;Bbs4-/- and Mks1krc/krc;Ift172avc1/avc1 double mutant cells as well as WT and single mutant cells. Expression was analyzed with three biological replicates for each experimental condition, with three technical replicates for each biological replicate. The graphs depict the average relative Gli2 levels (normalized to GAPDH for each replicate), error bars are standard deviation. (TIF) [file pone.0173399.s002.tif]

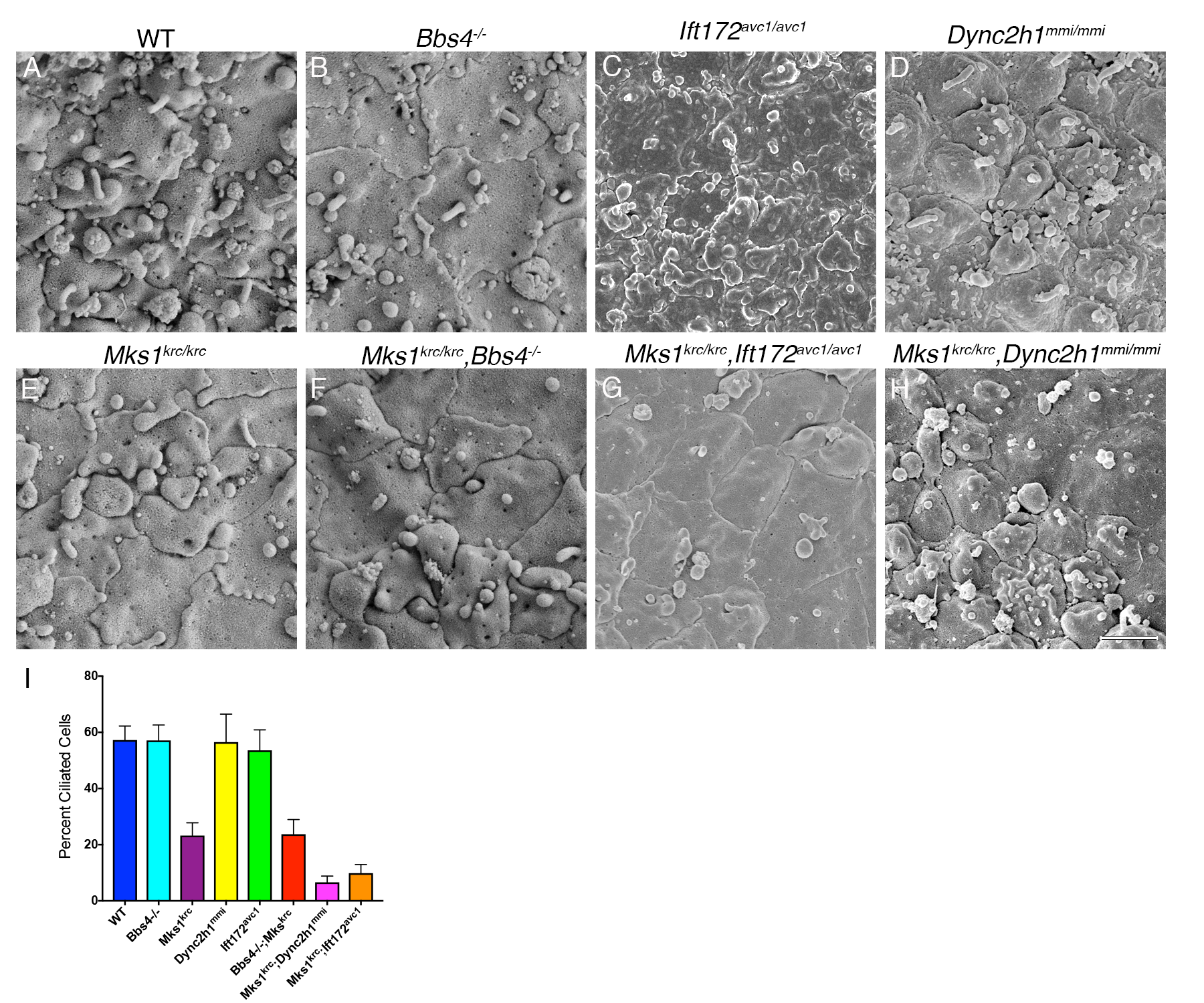

Supplement: S3 Fig — (A-H) Embryos of the indicated genotypes were collected at E10.5, and the neural tubes were dissected out and fixed. Neural tubes were then opened and imaged en face by SEM. The regions imaged correspond to the ventral portion of the neural tube, anterior to the forelimbs. Scale bar, 1μm. (I) Quantification of the percentage of cells that are ciliated within the neural tube for each genotype. E10.5 SEM images of the neural tube were imaged as in A-H. A minimum of 2 fields were imaged for each of 3 embryos for each genotype at the forelimb level. In each field, cells with clear boundaries were counted and scored for whether or not they had a cilium. The percentage of ciliated cells for each field was recorded. Bars represent the mean of all fields examined for each genotype, and error bars are standard deviation. Genotypes were compared using ANOVA with a Tukey-Kramer multiple-comparison correction. Similar to our data based on percentage of ciliated cells in MEFs: we find that cilia frequency in Bbs4-/-, Ift172avc1/avc1, and Dync2h1mmi/mmi single mutants is comparable to WT embryos. Mks1krc/krc;Bbs4-/- have similar numbers of cilia to Mks1krc/krc single mutants (p>0.99), however both Mks1krc/krc; Dync2h1mmi/mmi and Mks1krc/krc;Ift172avc1/avc1 double mutants have significantly fewer cilia than Mks1krc/krc single mutants (p<0.0001, and p = 0.0002, respectively). (TIF) [file pone.0173399.s003.tif]

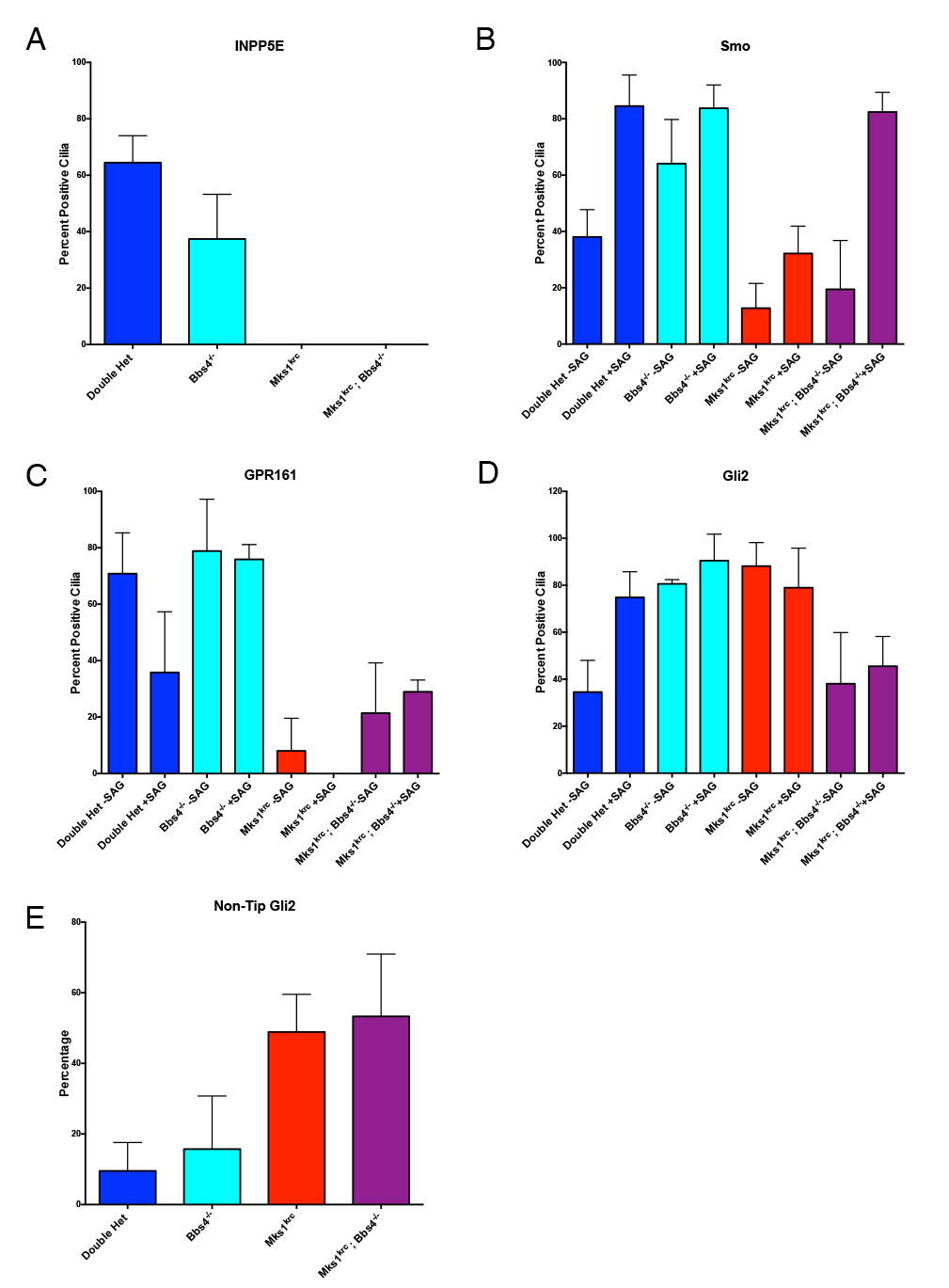

Supplement: S4 Fig — Related to Fig 3. (A-D) Each graph shows the percentage of cilia positive for the indicated marker. Bars represent the mean percent positive cilia for each genotype, error bars represent standard deviation. (E) The graph represents the percentage of GLI2+ cilia for each genotype (upon SAG treatment) in which GLI2 was not restricted to the ciliary tip (extending more than 1/3 of the length of the cilium from the tip, or seen in a location of the cilium other than the tip such as the base). Bars represent the mean percent of GLI2+ cilia with non-tip GLI2, error bars represent standard deviation. (TIF) [file pone.0173399.s004.tif]

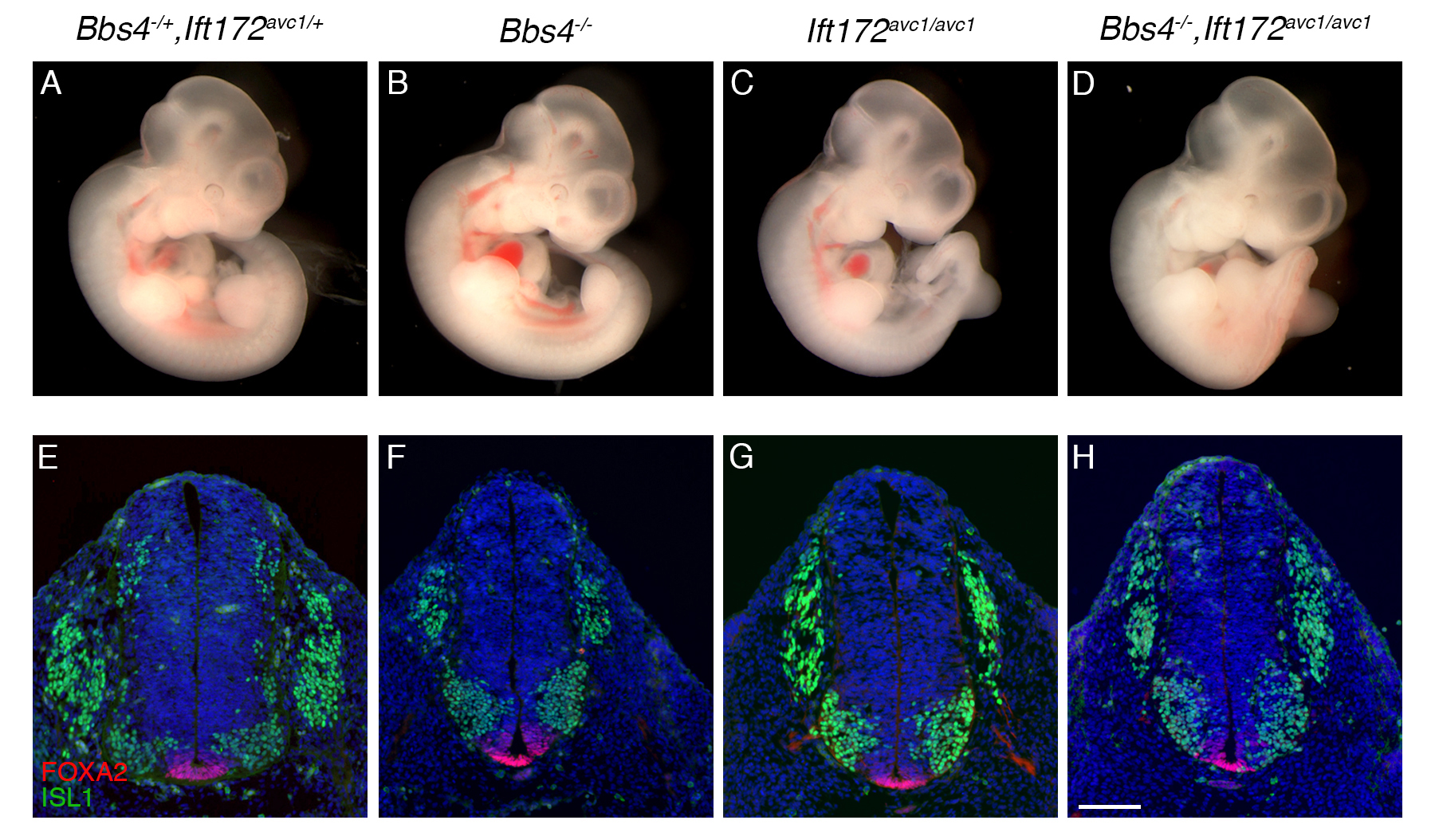

Supplement: S5 Fig — (A-D) Double heterozygous (A), Bbs4-/- (B), Ift172avc1/avc1 (C), or Ift172avc1/avc1;Bbs4-/- double mutant (D) embryos at E10.5. (E-H) Transverse sections through the neural tube of embryos of the indicated genotype at E10.5. Sections were taken at the level of the forelimbs and immunostained with antibodies against FOXA2 (red) and ISL1 (green). Scale bar, 100μm. (TIF) [file pone.0173399.s005.tif]
