# Supplementary material for: The Meckel syndrome- associated protein MKS1 functionally interacts with components of the BBSome and IFT complexes to mediate ciliary trafficking and hedgehog signaling
Source: PLoS One. 2017 Mar 14;12(3):e0173399. doi: 10.1371/journal.pone.0173399 (PMC5349470; doi:10.1371/journal.pone.0173399)
Supplement: S1 Table — (DOCX) [file pone.0173399.s006.docx]

**Table S1. Total numbers of embryos recovered for each genotype in double mutant crosses.**

Mks1xIft172

Total embryos: 266. Analyzed at E10.5

| Genotype | +/+ | m+/+;i+/- | m+/-;i+/+ | m+/-;I+/- | m-/-;i+/+ | m-/-;i+/- | m+/+;i-/- | m+/-;i-/- | m-/-;i-/- |
| --- | --- | --- | --- | --- | --- | --- | --- | --- | --- |
| Total Number | 22 | 32 | 40 | 63 | 15 | 31 | 13 | 41 | 9 |
| Percent | 8.27 | 12.03 | 15.04 | 23.68 | 5.64 | 11.65 | 4.89 | 15.41 | 3.38 |

Mks1xBbs4

Total embryos: 276. Analyzed at E10.5-E14.5

| Genotype | +/+ | m+/+;b+/- | m+/-;b+/+ | m+/-;b+/- | m-/-;b+/+ | m-/-;b+/- | m+/+;b-/- | m+/-;b-/- | m-/-;b-/- |
| --- | --- | --- | --- | --- | --- | --- | --- | --- | --- |
| Total Number | 20 | 32 | 28 | 75 | 12 | 35 | 19 | 31 | 24 |
| Percent | 7.25 | 11.59 | 10.14 | 27.17 | 4.35 | 12.68 | 6.88 | 11.23 | 8.70 |

Mks1xDyn

Total embryos: 103. Analyzed at E10.5

| Genotype | +/+ | m+/+;d+/- | m+/-;d+/+ | m+/-;d+/- | m-/-;d+/+ | m-/-;d+/- | m+/+;d-/- | m+/-;d-/- | m-/-;d-/- |
| --- | --- | --- | --- | --- | --- | --- | --- | --- | --- |
| Total Number | 2 | 15 | 15 | 21 | 11 | 10 | 7 | 13 | 9 |
| Percent | 1.94 | 14.56 | 14.56 | 20.34 | 10.68 | 9.71 | 6.80 | 12.62 | 8.73 |

Bbs4xIft172

Total embryos: 227. Analyzed at E10.5

| Genotype | +/+ | b+/+;i+/- | b+/-;i+/+ | b+/-;i+/- | b-/-;i+/+ | b-/-;i+/- | b+/+;i-/- | b+/-;i-/- | b-/-;i-/- |
| --- | --- | --- | --- | --- | --- | --- | --- | --- | --- |
| Total Number | 13 | 19 | 33 | 72 | 17 | 20 | 8 | 30 | 15 |
| Percent | 5.73 | 8.37 | 14.54 | 31.72 | 7.49 | 8.81 | 3.52 | 13.22 | 6.61 |

Abbreviations: m=Mks1^krc^; i=Ift172^avc1^; b=Bbs4; d=Dync2h1^mmi^, += WT allele, -= mutant allele.
